# Asymmetric Cu(I)─W Dual‐Atomic Sites Enable C─C Coupling for Selective Photocatalytic CO_2_ Reduction to C_2_H_4_

**DOI:** 10.1002/advs.202401933

**Published:** 2024-04-26

**Authors:** Yuyin Mao, Minghui Zhang, Guangyao Zhai, Shenghe Si, Dong Liu, Kepeng Song, Yuanyuan Liu, Zeyan Wang, Zhaoke Zheng, Peng Wang, Ying Dai, Hefeng Cheng, Baibiao Huang

**Affiliations:** ^1^ State Key Laboratory of Crystal Materials Shandong University Jinan 250100 China; ^2^ School of Chemistry and Materials Science University of Science and Technology of China Hefei 230026 China; ^3^ School of Chemistry and Chemical Engineering Shandong University Jinan 250100 China; ^4^ School of Physics Shandong University Jinan 250100 China

**Keywords:** asymmetric dual sites, C_2_H_4_ generation, C─C coupling, CO_2_ photoreduction, Cu(I) single atoms

## Abstract

Solar‐driven CO_2_ reduction into value‐added C_2+_ chemical fuels, such as C_2_H_4_, is promising in meeting the carbon‐neutral future, yet the performance is usually hindered by the high energy barrier of the C─C coupling process. Here, an efficient and stabilized Cu(I) single atoms‐modified W_18_O_49_ nanowires (Cu_1_/W_18_O_49_) photocatalyst with asymmetric Cu─W dual sites is reported for selective photocatalytic CO_2_ reduction to C_2_H_4_. The interconversion between W(V) and W(VI) in W_18_O_49_ ensures the stability of Cu(I) during the photocatalytic process. Under light irradiation, the optimal Cu_1_/W_18_O_49_ (3.6‐Cu_1_/W_18_O_49_) catalyst exhibits concurrent high activity and selectivity toward C_2_H_4_ production, reaching a corresponding yield rate of 4.9 µmol g^−1^ h^−1^ and selectivity as high as 72.8%, respectively. Combined in situ spectroscopies and computational calculations reveal that Cu(I) single atoms stabilize the *CO intermediate, and the asymmetric Cu─W dual sites effectively reduce the energy barrier for the C─C coupling of two neighboring CO intermediates, enabling the highly selective C_2_H_4_ generation from CO_2_ photoreduction. This work demonstrates leveraging stabilized atomically‐dispersed Cu(I) in asymmetric dual‐sites for selective CO_2_‐to‐C_2_H_4_ conversion and can provide new insight into photocatalytic CO_2_ reduction to other targeted C_2+_ products through rational construction of active sites for C─C coupling.

## Graphical abstract

An efficient Cu(I) single atoms‐modified W_18_O_49_ (Cu_1_/W_18_O_49_) photocatalyst is rationally designed for photocatalytic CO_2_ reduction to C_2_H_4_. Compared to W_18_O_49_ that produces CO only, Cu(I) single atoms stabilize *CO intermediates and the asymmetric Cu─W dual sites significantly reduce the energy barrier of C─C coupling, thus leading to the highly selective C_2_H_4_ generation from CO_2_ photoreduction in Cu_1_/W_18_O_49_.

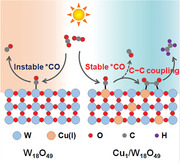

## 1 Introduction

Solar‐driven conversion of CO_2_ and water into high‐value‐added chemical fuels represents a promising strategy to mitigate the global greenhouse effect and fossil energy crisis.^[^ ^1^, ^2^, ^3^, ^4^, ^5^, ^6^ ^]^ As photocatalytic CO_2_ reduction comprises multiple proton‐coupled electron transfer (PCET) processes, a variety of carbon‐containing products, including CO, CH_3_OH, CH_4_, and even advanced hydrocarbons, are thus obtained. Due to the favorable kinetics, C_1_ compounds such as CO and CH_4_ dominate the products of photocatalytic CO_2_ reduction in most cases.^[^ ^7^, ^8^, ^9^, ^10^ ^]^ By contrast, C_2+_ compounds generated by the C─C coupling are considered to be more intriguing because of their high energy density and rich chemical reactivity. Of particular attention has been paid to the C_2_H_4_ product, which is a highly important chemical raw material that is widely used in rubber industry, medicine, and agriculture.^[^ ^11^, ^12^, ^13^ ^]^ However, the high energy barrier of the C─C coupling make it rather difficult to achieve C_2_H_4_ production with concurrent high activity and selectivity from photocatalytic CO_2_ reduction.^[^ ^14^, ^15^, ^16^ ^]^

Among CO_2_ reduction catalysts, Cu‐based catalysts are of great interest for their excellent ability to produce C_2_H_4_ and other C_2+_ products.^[^ ^17^, ^18^, ^19^, ^20^ ^]^ In electrocatalysis, Cu species, especially in the form of residual Cu(I), are considered as the most thermodynamically‐favorable catalysts for C─C coupling,^[^ ^21^, ^22^, ^23^ ^]^ where appropriate binding strength to the *CO intermediate facilitates their subsequent coupling process.^[^ ^24^, ^25^, ^26^, ^27^ ^]^ For photocatalytic process, however, the valence state of Cu(I) species (Cu_2_O) is variable, which could be reduced to metallic Cu^0^ by photogenerated electrons or oxidized to Cu(II) by photogenerated holes, thus leading to the unwanted deactivation. Consequently, it is ideal to develop and maintain stable Cu(I) active sites during photocatalytic CO_2_ reduction. Moreover, the production of C_2_H_4_ necessitates the coupling of adsorbed CO molecules at two neighboring sites to generate the key *CO─CO intermediate.^[^ ^28^, ^29^ ^]^ Nevertheless, it becomes quite difficult to perform C─C coupling in conventional semiconductor photocatalysis, where almost identical charge distribution between symmetric double Cu sites inevitably results in strong dipole‐dipole repulsion, thereby improving the energy barrier for C─C coupling. Alternatively, design of photocatalyst systems with asymmetric dual sites that are able to attenuate the dipole repulsion is more appealing.^[^ ^30^, ^31^ ^]^ Therefore, it is of paramount importance to develop stable and asymmetric Cu(I)‐containing dual sites during photocatalytic CO_2_ reduction, which could accelerate C─C coupling process toward C_2_H_4_ formation, and meanwhile this is rather challenging at the atomic level.

The unique crystal structure enables W_18_O_49_ to possess a high concentration of lattice defects and distortions, thereby providing the increased doping capacity of heteroatom atoms (e.g., Cu atoms).^[^ ^32^, ^33^ ^]^ Furthermore, according to the standard electrode potential, the abundant W^5+^ ions in W_18_O_49_ can reduce Cu^2+^ ions to Cu^+^ ions (Equation 1).^[^ ^34^ ^]^ As a consequence, the interconversion between W(V) and W(VI) in W_18_O_49_ could ensure the stability of Cu(I) during the photocatalytic process.

(1) $$W^{5+}+Cu^{2+}\to W^{6+}+Cu^{+}$$

Guided by the two features above, the defect‐rich W_18_O_49_ ultrathin nanowires were chosen as an ideal support for construction of stable and asymmetric Cu(I)‐containing dual sites. To this end, through rational incorporation of Cu(I) single atoms in W_18_O_49_ nanowires (Cu_1_/W_18_O_49_), we have reported here the realization of photocatalytic CO_2_ reduction to C_2_H_4_ with concurrent high activity and selectivity. The interconversion between W(V) and W(VI) in W_18_O_49_ ensures the stability of Cu(I) during the photocatalytic process, and the resulting asymmetric Cu─W dual sites were thus constructed to stabilize *CO intermediates and promote C─C coupling. At an optimal concentration, Cu_1_/W_18_O_49_ catalyst exhibits a high activity (4.9 µmol g^−1^ h^−1^) and high selectivity (72.8%) for CO_2_ photoreduction toward C_2_H_4_ production. In situ near‐ambient pressure X‐ray photoelectron spectroscopy (NAP‐XPS) and diffuse reflectance infrared Fourier‐transform spectroscopy (DRIFTS) measurements were carried out to elucidate the transfer direction of photoinduced charge, decipher the catalytically‐active sites and reveal the reaction path for photocatalytic CO_2_ reduction to C_2_H_4_. The detailed experimental characterizations and density functional theory (DFT) calculations unravel that both Cu(I) and W(V) atoms are enriched in electrons as likely active sites for photocatalytic CO_2_ reduction toward C_2_H_4_ evolution, and the asymmetric Cu─W dual sites effectively reduce the energy barrier for the coupling of two neighboring CO intermediates.

## 2 Results and Discussion

### 2.1 Synthesis and Characterizations

**Figure 1:**
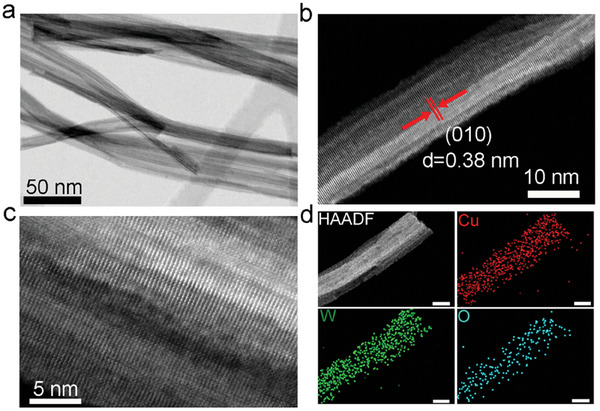
Morphological characterizations. a,b) Low‐magnification HAADF‐STEM images of 3.6‐Cu_1_/W_18_O_49_. c) Atomic resolution HAADF‐STEM image of 3.6‐Cu_1_/W_18_O_49_. d) EDX mapping images of Cu (red), W (green), and O (cyan) in 3.6‐Cu_1_/W_18_O_49_ nanowire. The scale bars are 5 nm.

The Cu‐modified W_18_O_49_ catalysts with different Cu loadings (*x* wt.%) were synthesized by a one‐step hydrothermal method (see details in the Supporting Information), and the resulting samples were designated as *x*‐Cu_1_/W_18_O_49_ (*x* = 1.4−7.0). The actual concentration of Cu in various Cu_1_/W_18_O_49_ samples was measured by inductively coupled plasma mass spectrometry (ICP‐MS) and found to be in the range of 1.29−5.01 wt.% (Table S1, Supporting Information). As shown in the X‐ray diffraction (XRD) patterns (Figure S1, Supporting Information), all samples are well indexed to monoclinic W_18_O_49_ (JCPDS No. 36–101) phase, and no diffraction peaks of Cu‐containing species (i.e., Cu and CuO_x_) are observed. Additionally, as Cu loading increases, the spacing of characteristic W_18_O_49_ (020) crystal plane becomes widened with a decreased signal‐to‐noise ratio, indicating the successful doping of Cu into the lattice of W_18_O_49_ host material.^[^ ^35^ ^]^ The morphological information of the samples was revealed by scanning electron microscopy (SEM, Figure S2, Supporting Information) and transmission electron microscopy (TEM, Figure S3, Supporting Information). Analogously to pristine W_18_O_49_, Cu‐modified W_18_O_49_ samples consist of homogeneous nanowires with lengths of 200−500 nm and diameters of 5−10 nm, and no aggregated nanoparticles are observed on the surfaces. To further reveal the distribution and configuration of Cu species in Cu_1_/W_18_O_49_, high‐angle annular dark‐field scanning transmission electron microscopy (HAADF‐STEM) with atomic resolution was carried out. From the low‐resolution HAADF‐STEM images of 3.6‐Cu_1_/W_18_O_49_ sample (**Figure** **1**a,b), well‐defined nanowires with a lattice spacing of 0.38 nm are observed, corresponding well to the (010) crystal plane spacing of monoclinic W_18_O_49_, indicating that the nanowires grow along the [010] direction. As shown in Figure 1c, no clusters or nanoparticles of Cu species are found in the HAADF‐STEM image at atomic resolution, verifying that Cu species are highly dispersed at the atomic level in W_18_O_49_ nanowires. Additionally, nanometer‐resolution energy‐dispersive X‐ray (EDX) elemental mapping (Figure 1d) displays that Cu, W, and O are uniformly distributed in the nanowire, further confirming the atomically‐dispersed Cu in the W_18_O_49_ structure.

**Figure 2:**
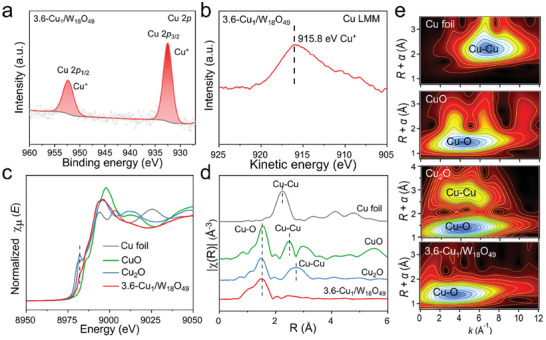
Structural characterizations of Cu_1_/W_18_O_49_. a) Cu 2*p* XPS spectra and b) Cu LMM Auger spectrum of Cu_1_/W_18_O_49_. c) Normalized Cu K‐edge XANES spectra, d) FT‐EXAFS spectra. e) WT‐EXAFS of Cu K‐edge for Cu_1_/W_18_O_49_, Cu foil, Cu_2_O, and CuO references.

To elucidate the oxidation state of Cu species and its interaction with the W_18_O_49_ support, high‐resolution X‐ray photoelectron spectra (XPS, **Figure** **2**a) were carried out. As presented in Figure 2a, the primary Cu 2*p*_3/2_ peak was observed to be located at a binding energy of 932.0 eV, corresponding well to the monovalent Cu(I) ions.^[^ ^36^, ^37^ ^]^ Additionally, the existence of Cu(II) is excluded by the absence of its characteristic satellite peaks. As shown in the Cu LMM Auger spectrum (Figure 2b), only a characteristic peak for Cu(I) at the kinetic energy of 915.8 eV is detected, while the characteristic peak for Cu^0^ at 918.3 eV is absent.^[^ ^38^, ^39^ ^]^ The results above indicate that the Cu^2+^ ions added to the precursor solution were reduced to Cu(I) anchored on the W_18_O_49_ nanowires. According to the standard electrode potential, W(V) can reduce Cu^2+^ ions to Cu^+^ ions (Equation 1), but it cannot be determined whether W(V) can reduce Cu^2+^ to Cu(I) sites. However, it can be seen from the XPS spectrum of W 4*f* (Figure S4, Supporting Information) that the introduction of Cu single atoms significantly reduces the content of W^5+^ from 26.12% to 23.55%, indicating that W(V) can reduce Cu^2+^ to Cu(I) sites. This result also indicates that existence of a fast electron transfer channel between Cu(I) sites and W atoms. At the same time, due to the decrease of W^5+^ content, a decrease in the visible light absorption of Cu_1_/W_18_O_49_ is seen compared to pristine W_18_O_49_ (Figure S5, Supporting Information). As a consequence, the Cu species in Cu_1_/W_18_O_49_ can be slightly reduced by electrons from W(V) of W_18_O_49_ and thus maintain its oxidation state of +1.

To further determine the chemical environment and coordination structure of atomically‐dispersed Cu species in Cu_1_/W_18_O_49_, Cu K‐edge X‐ray absorption near‐edge structure (XANES) and extended X‐ray absorption fine structure (EXAFS) spectra were performed. As shown in Figure 2c, in contrast to CuO and Cu foil, the normalized Cu K‐edge XANES spectrum of 3.6‐Cu_1_/W_18_O_49_ exhibits an absorption edge more close to Cu_2_O reference. From the linear fitting for the peak position of the first derivative in XANES curves (Figure S6, Supporting Information), it is verified that the valence state of Cu species in Cu_1_/W_18_O_49_ is located ≈+1, and this finding is in good line with the XPS results (Figure 2a,b). rom the Fourier transform EXAFS (FT‐EXAFS) spectra (Figure 2d), 3.6‐Cu_1_/W_18_O_49_ displays a dominant peak belonging to the Cu─O bond at ≈1.5 Å. Compared to Cu‐based references, either the Cu─Cu bond (2.25 Å for Cu foil) or Cu─O─Cu bond (2.45 Å for CuO and 2.72 Å for Cu_2_O) is absent in Cu_1_/W_18_O_49_, suggesting an isolated distribution of Cu atoms in W_18_O_49_ nanowires. The accurate coordination structure of Cu_1_/W_18_O_49_ was obtained through least‐squares fitting of the K‐edge EXAFS data (Figure S7 and Table S2, Supporting Information). Notably, the fitting results reveal a Cu─O coordination number of 3.89 in 3.6‐Cu_1_/W_18_O_49_ nanowires, indicating that Cu atoms are predominantly anchored on the surface of W_18_O_49_ and coordinated by oxygen atoms. This result is in good accordance with the electron spin resonance (ESR, Figure S8, Supporting Information) test, where the concentration of oxygen vacancies (*g* = 2.003) gradually decreases with an increase in Cu doping ratio, probably due to the occupation of oxygen vacancies by a significant amount of atomically‐dispersed Cu sites.^[^ ^40^ ^]^ Apart from Cu─O bonds, 3.6‐Cu_1_/W_18_O_49_ also shows a considerable portion of Cu─W path with a coordination number of 0.88 from the fitted second shell layer. The wavelet transform (WT) analysis was performed to obtain more intuitive data on atom distance (Figure 2e). The WT intensity maxima of Cu_1_/W_18_O_49_ is observed ≈4.0 Å^−1^, which overlaps with the Cu─O bond of Cu_2_O reference. In contrast, the intensity maximum of Cu foil at 6.7 Å^−1^ ascribed to the Cu─Cu bond is not observed in Cu_1_/W_18_O_49_, further confirming the presence of atomically isolated Cu species in Cu_1_/W_18_O_49_.

### 2.2 Photocatalytic CO_2_ Reduction Evaluation

**Figure 3:**
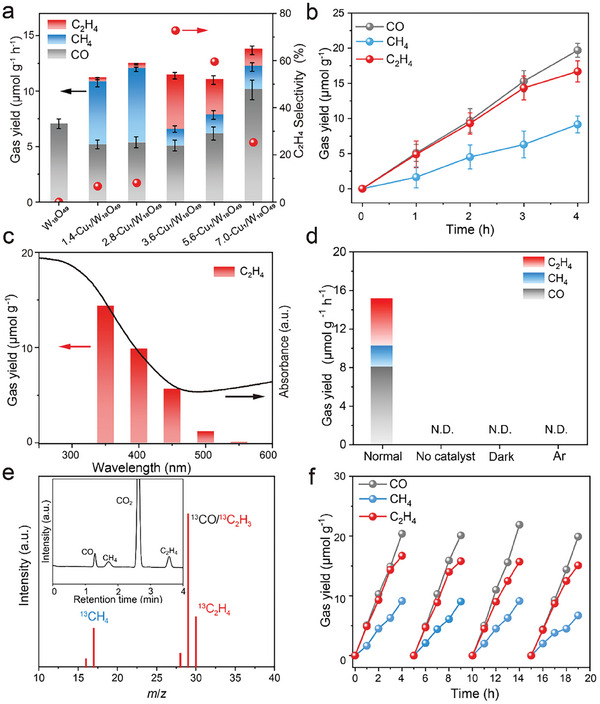
Photocatalytic CO_2_ reduction performance evaluation. a) The yield rate and selectivity of products over different catalysts. b) The yield rate products as a function of reaction time over 3.6‐Cu_1_/W_18_O_49_. c) The measured wavelength‐dependent gas yield rates with monochromatic incident light irradiation for 4 h. d) The control experiments of photocatalytic CO_2_ reduction performances under different conditions, where N.D. denotes product not detected. e) MS spectrum of ^13^C_2_H_4_ (*m*/*z* = 30) production from photocatalytic ^13^CO_2_ reduction over 3.6‐Cu_1_/W_18_O_49_. Inset: the corresponding GC spectrum. f) Cycling measurements for CO_2_ photoreduction.

The photocatalytic CO_2_ reduction performance of the as‐prepared samples were evaluated in a solid‐gas system using a 300 W Xe lamp as the light source without any sacrificial agent (Figure S9, Supporting Information). The gaseous products were monitored and quantified by gas chromatography (GC, Figure S10, Supporting Information). In the case of pristine W_18_O_49_, only CO product was obtained with a yield rate of 7.1 µmol g^−1^ h^−1^ (**Figure** **3**a). Interestingly, the introduction of atomically‐dispersed Cu sites improves the photocatalytic activity and selectivity, along with the production of CH_4_ and C_2_H_4_. This suggests that Cu single atoms could stabilize the adsorption of CO and facilitate its subsequent PCET process to generate hydrocarbons. For 1.4‐Cu_1_/W_18_O_49_ sample, apart from CO production, it exhibited CH_4_ and C_2_H_4_ production rates of 5.7 and 0.33 µmol g^−1^ h^−1^, respectively, with a low selectivity of 6.7% for C_2_H_4_ product. At an optimal doping concentration of Cu single atoms, C_2_H_4_ production reaches the maximum over 3.6‐Cu_1_/W_18_O_49_ photocatalyst. As shown in Figure 3b, 3.6‐Cu_1_/W_18_O_49_ photocatalyst exhibited a near‐linear increase of gas phase products with prolonged light irradiation time, and could deliver a yield rate of 4.9 µmol g^−1^ h^−1^ for C_2_H_4_ product, along with considerable production of CO (5.1 µmol g^−1^ h^−1^) and CH_4_ (2.2 µmol g^−1^ h^−1^). In addition, O_2_ as the oxidation product was detected by GC and found to increase linearly with the reaction time (Figure S11, Supporting Information). Accordingly, a high selectivity of 82.5% for hydrocarbons (CH_4_ + C_2_H_4_) and 72.8% for C_2_H_4_ is thus reached over 3.6‐Cu_1_/W_18_O_49_ photocatalyst. Notably, in this work, 3.6‐Cu_1_/W_18_O_49_ photocatalyst exhibits a satisfactory concurrent high activity and high selectivity of C_2_H_4_ production from solar‐driven CO_2_ reduction, outperforming most of the reported Cu(I)‐based photocatalysts (Table S3, Supporting Information) and being comparable to those state‐of‐the‐art photocatalysts systems toward C_2+_ products (Table S4, Supporting Information). Further increase of Cu doping concentrations, however, leads to the decreased activity and selectivity of C_2_H_4_ product over 5.6‐Cu_1_/W_18_O_49_ (3.7 µmol g^−1^ h^−1^, 64.4%) and 7.0‐Cu_1_/W_18_O_49_ (1.53 µmol g^−1^ h^−1^, 25.3%) photocatalysts, which could be probably due to their inferior separation capacity of photogenerated carriers (Figure S12, Supporting Information).

The wavelength‐dependent experiments were carried out with monochromatic incident light irradiation. As shown in Figure 3c, the yield rate of C_2_H_4_ product from photocatalytic CO_2_ reduction corresponds well to the light absorption of 3.6‐Cu_1_/W_18_O_49_ photocatalyst, giving direct proof that CO_2_ reduction is driven by solar energy. To exclude the possible carbon contamination, a series of controlled experiments were conducted. From Figure 3d, it demonstrates that almost no gas‐phase products were detected in the absence of light irradiation, CO_2_ feeding gas, or Cu_1_/W_18_O_49_ photocatalyst, thus verifying the C_2_H_4_ product is derived from light‐driven photocatalytic CO_2_ reduction process on Cu_1_/W_18_O_49_. Furthermore, isotope labeling experiments using ^13^CO_2_ feeding gas were conducted to verify the origin of carbon in the product. As depicted in Figure 3e, along with the GC spectrum, peaks of ^13^C_2_H_4_ (*m*/*z* = 30), ^13^CO *(m*/*z* = 29), and ^13^CH_4_ (*m*/*z* = 17) were detected in the mass spectrometry (MS) spectrum, confirming that the products resulted from CO_2_ reduction rather than possible carbon contamination. Apart from activity and selectivity, stability is another important factor in assessing the performance of photocatalysts. To demonstrate the stability of Cu(I), the Auger spectra of Cu_1_/W_18_O_49_ after photocatalytic CO_2_ reaction was also measured (Figure S13, Supporting Information). As expected, the oxidation state of Cu in Cu_1_/W_18_O_49_ remained +1 after photocatalytic CO_2_ reaction, confirming the effective stabilization of Cu(I). Moreover, after four consecutive photocatalytic cycles, 3.6‐Cu_1_/W_18_O_49_ photocatalyst maintained nearly constant activity (Figure 3f) and unaltered structure (Figures S14–S16, Supporting Information), demonstrating its good stability in both performance and structure.

### 2.3 Active Sites Identification

**Figure 4:**
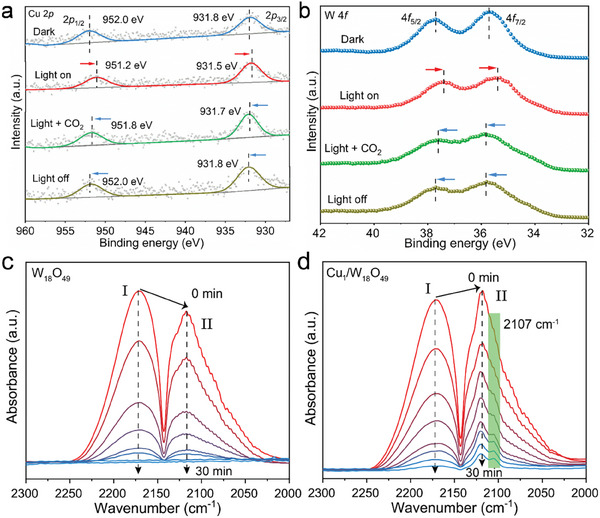
Active sites identification. a) Cu 2*p* and b) W 4*f* in situ NAP‐XPS spectra obtained over 3.6‐Cu_1_/W_18_O_49_ photocatalyst. In‐situ DRIFTS spectra obtained during CO desorption on c) W_18_O_49_ and d) Cu_1_/W_18_O_49_ under Ar purging at room temperature and ambient pressure.

To understand the high activity and selectivity of C_2_H_4_ production over 3.6‐Cu_1_/W_18_O_49_ photocatalyst for CO_2_ reduction, a comprehensive study was conducted to decipher the possible active sites, especially the C─C coupling sites. To this end, in situ NAP‐XPS measurements were performed to illustrate the transfer direction of photoinduced charges and identify the probable active site during photocatalysis. As shown in **Figure** **4**a, in clear contrast to dark condition, a dramatic shift of the Cu(I) peak (952.0 eV) to lower binding energy (951.2 eV) can be observed upon light irradiation, indicating that Cu(I) single atoms are enriched with photoinduced‐electrons. More importantly, when CO_2_ gas is introduced into the system, the peak of Cu(I) shifts back toward higher binding energy (951.8 eV), which indicates that Cu(I) ions lose partial electrons by donating them to the surface‐adsorbed CO_2_ molecules.^[^ ^41^, ^42^ ^]^ In addition, after removing the light irradiation, Cu(I) single atoms recover to their original state at 952.0 eV and in such way Cu(I) single atoms maintain stability after the reaction. According to previous studies,^[^ ^41^, ^43^, ^44^ ^]^ the electron transfer processes under illumination involve two main stages: a rapid accumulation of photogenerated electrons and a slower chemical reduction process. The former typically occurs within seconds and importantly, it is reversible compared to the latter. In our test, we clearly observe the sample returning to its original state after the illumination ends, indicating that only the reversible first stage of photogenerated electron accumulation on Cu(I) occurs under illumination, and there is no chemical reduction of Cu(I) during this process. This observation further supports the stability of Cu(I) single atoms during photocatalytic reactions. Analogously, a similar phenomenon is observed in the in‐situ NAP‐XPS spectra of W 4*f* in 3.6‐Cu_1_/W_18_O_49_ (Figure 4b). Compared to dark condition, light irradiation enables the W atoms to capture photogenerated electrons to give birth to W(V) ions with a lower valence state. Generally, the Cu_1_/W_18_O_49_ catalyst contains two types of W atoms, that is, one close to the Cu single atoms to form Cu─W dual sites and one far from the Cu single atoms. The electron transfer in the light of the specific two W atoms can be further determined by the product distribution of photocatalytic CO_2_ reduction. Specifically, W atoms far from the Cu single atoms can only generate CO, while the W sites near the Cu single atoms can form asymmetric Cu─W dual sites to produce C_2_H_4_. The simultaneous production of CO and C_2_H_4_ in 3.6‐Cu_1_/W_18_O_49_ indicates that both W atoms are enriched in electrons for CO_2_ reduction. Upon further introduction of CO_2_ gas, W(V) ions are partially re‐oxidized to W(VI) ions by transferring electrons to surface‐adsorbed CO_2_ molecules, and eventually only a portion of W atoms recover to their original state when the light irradiation is off. As a result, the stability of Cu(I) can be ensured by the following reasons: i) The abundant W(V) in W_18_O_49_, which is more easily oxidized, can reduce Cu(II) to Cu(I) to avoid its oxidation; ii) Under light irradiation, the more easily reduced W(VI) around Cu(I) inhibits the excessive aggregation of photo‐generated electrons, thus avoiding the reduction of Cu(I) to Cu(0). Therefore, the coexistence of W(V) and W(VI) ensures the stability of Cu(I) sites during photocatalytic process. In summary, in‐situ NAP‐XPS measurement results indicate that both Cu(I) and W(V) atoms are probable active sites for photocatalytic CO_2_ reduction toward C_2_H_4_.

To further determine the active sites of C─C coupling, we conducted in‐situ DRIFTS experiments using CO as a probe molecule, which is a key intermediate of CO_2_ reduction to C_2_H_4_. In the test, the catalyst was initially exposed to CO gas adsorption until saturation and subsequently treated with Ar gas to desorb the physically‐adsorbed CO while retaining the chemically‐adsorbed CO. As shown in the DRIFT spectra of adsorbed CO on pristine W_18_O_49_ (Figure 4c) and 3.6‐Cu_1_/W_18_O_49_ (Figure 4d) catalysts reveal two bands centered at 2170 and 2120 cm^−1^. Generally, the peak located at 2170 cm^−1^ represents physically adsorbed CO (mode‐I),^[^ ^45^, ^46^ ^]^ where the adsorption peak in the range of 2150−2100 cm^−1^ indicates more complex CO adsorption behavior (mode‐II).^[^ ^47^ ^]^ It is worth noting that the peak intensity of mode‐I is higher than that of mode‐II over pristine W_18_O_49_, while the peak intensity of mode‐II is higher than that of mode‐I after the introduction of Cu(I) single atoms in 3.6‐Cu_1_/W_18_O_49_, confirming that Cu(I) single atoms indeed enhance the adsorption of CO. After Ar purging, both absorption peaks gradually weaken, and the CO absorption peak of W_18_O_49_ disappeared completely after 30 min, indicative of only weak physically‐adsorbed CO on W_18_O_49_. Intriguingly, following a long purge process, the mode‐I adsorption on 3.6‐Cu_1_/W_18_O_49_ was completely eradicated, while mode‐II adsorption remains and can be further divided into two individual peaks located at 2120 and 2107 cm^−1^, respectively. According to previous studies, the peak at 2107 cm^−1^ is the characteristic peak of the linear adsorption of CO on Cu(I) sites.^[^ ^48^, ^49^, ^50^ ^]^ Due to the absence of other oxidation states of Cu species in Cu_1_/W_18_O_49_ as CO adsorption sites, the peak at 2120 cm^−1^ is more likely attributed to CO chemisorption on W atoms. The CO adsorption free energy further supports this observation (Table S5, Supporting Information), as introducing Cu atoms reduce the CO adsorption energy on W atoms from −0.14 eV in W_18_O_49_ to −0.52 eV in Cu_1_/W_18_O_49_. It is thus concluded that Cu(I) sites not only have a stronger adsorption strength of CO, but they stabilize the adsorbed CO on W atoms. This result also indicates that the W sites in the Cu─W dual sites can also have a stronger adsorption of *CO. The simultaneous enhancement of *CO adsorption strength in the Cu─W dual sites is conducive to the generation of two adjacent *CO, thereby accelerating the subsequent C─C coupling process. In addition, the disparity in the adsorption of CO on W_18_O_49_ and 3.6‐Cu_1_/W_18_O_49_ was probed using CO temperature‐programmed desorption (CO‐TPD) analysis (Figure S17, Supporting Information). Pristine W_18_O_49_ exhibited two desorption peaks, that is, the weak (200−230 °C) and strong CO adsorption (350−380 °C), in the range of 100−600 °C. However, after the introduction of Cu(I) single atoms, both of the desorption peaks shifted toward higher temperatures, indicating that Cu(I) single atoms enhance the adsorption of CO.

To demonstrate the unique role of Cu(I) sites for CO adsorption, Cu(II)/WO_3_ sample that consists entirely of Cu(II) ions was prepared by calcining 3.6‐Cu_1_/W_18_O_49_ in air (see details in the Supporting Information). The corresponding characterizations (XRD and XPS, Figures S18 and S19, Supporting Information) showed that after calcination, W_18_O_49_ has been converted to WO_3_ and Cu(I) has been oxidized to Cu(II) ions. Different from 3.6‐Cu_1_/W_18_O_49_, Cu(II)/WO_3_ exhibited analogous CO adsorption behavior to pristine W_18_O_49_, where complete CO desorption takes place after Ar gas purge (Figure S20, Supporting Information). As a consequence, Cu(II)/WO_3_ displayed no production of C_2_H_4_ in the photocatalytic CO_2_ reduction test (Figure S21, Supporting Information), where only CO (10.1 µmol g^−1^ h^−1^) and a small amount of CH_4_ (1.4 µmol g^−1^ h^−1^) were produced. It is worth noting that the intensity and mode of CO significantly influence the energy barrier of C─C coupling and the selectivity of subsequent reduction products. Simultaneous adsorption of *CO molecules on adjacent Cu(I) and W(V) sites, that is asymmetric Cu─W dual sites, is an important process for the coupling of two CO molecules over the 3.6‐Cu_1_/W_18_O_49_ photocatalyst. Consistent with the fitted XAFS and in‐situ NAP‐XPS results, the DRIFT spectra for CO desorption provide further evidence supporting the existence of Cu─W dual sites.

### 2.4 Reaction Mechanism Investigation

The band structure of Cu_1_/W_18_O_49_ was first studied by Mott‐Schottky test (Figure S22a, Supporting Information). The flat band potential of 3.6‐Cu_1_/W_18_O_49_ was −0.32 V (vs NHE), and its conduction band (CB) position was estimated to be −0.62 V (vs NHE). For n‐type semiconductors, the CB position is ≈0.3 V lower than the flat‐band potential, so the CB position of 3.6‐Cu_1_/W_18_O_49_ is located at ≈−0.62 V (vs NHE), which is more negative than the reduction potential of CO_2_/CO (−0.51 V vs NHE), CO_2_/CH_4_ (−0.24 V vs NHE) and CO_2_/C_2_H_4_ (−0.33 V vs NHE). Apparently, it is indicated that the CB position of 3.6‐Cu_1_/W_18_O_49_ is thermodynamically favorable for photocatalytic CO_2_ reduction to carbon‐containing products of CO, CH_4_ and C_2_H_4_ (Figure S22b, Supporting Information).

**Figure 5:**
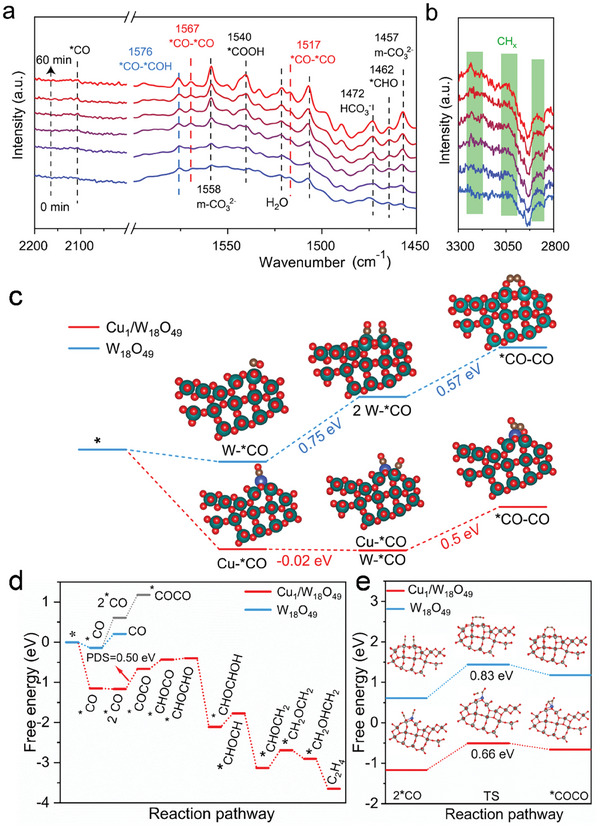
Reaction mechanism investigation. a) In situ DRIFTS detections on the Cu_1_/W_18_O_49_ photocatalyst in a humid CO_2_ atmosphere under light irradiation b) enlarged region for CH_x_ detection. c) Free energy changes of C─C coupling at different active sites. The corresponding structural model consists of Cu (blue), W (dark green), O (red) and C (gray) atoms. d) The free energy diagram of CO_2_ reduction on Cu_1_/W_18_O_49_ and W_18_O_49_. The blue line shows the more favorable way, while the gray line shows the less favorable way over W_18_O_49_ catalyst. e) Transition state energy barriers for C─C coupling on W_18_O_49_ and Cu_1_/W_18_O_49_.

To gain a deeper understanding of the CO_2_ photoreduction process, specifically the C─C coupling pathway, we conducted in‐situ DRIFTS measurements. As shown in **Figure** **5**a, a series of CO_2_ adsorption and reaction intermediates were monitored over the 3.6‐Cu_1_/W_18_O_49_ photocatalyst under light irradiation. The peak observed at 1472 cm^−1^ can be attributed to the asymmetric stretching vibrations of the HCO_3_^−^ group, while the peaks at 1457 and 1558 cm^−1^ are associated with the monodentate carbonate (m‐CO_3_^2−^) group.^[^ ^51^, ^52^ ^]^ In addition, the peaks observed at 1462, 1540, 2107, and 2120 cm^−1^ are attributed to the vibrations of *CHO, *COOH, and *CO groups, respectively, which are important intermediates in the production of CH_4_ and CO products.^[^ ^53^, ^54^ ^]^ Moreover, from the enlarged view in the range of 2200−2000 cm^−1^ (Figure S23, Supporting Information), two distinct peaks assigned to *CO intermediates can be clearly observed. Notably, the peaks at 1567 and 1517 cm^−1^ are assigned to asymmetric stretching vibrations and symmetric stretching vibrations of the *CO─*CO intermediate, respectively, which is key intermediate for the C─C coupling.^[^ ^55^, ^56^ ^]^ Importantly, we also observed the *CHO─CO intermediate (1576 cm^−1^) in the in‐situ DRIFT spectra that originates from the subsequent hydrogenation process of the *CO─*CO intermediate.^[^ ^57^ ^]^ In addition, broad peaks were observed at 3000−3300 cm^−1^ (Figure 5b), which are ascribed to C─H stretching vibration,^[^ ^58^ ^]^ suggesting the generation of hydrocarbons (i.e., CH_4_ and C_2_H_4_) in this work. By contrast, no clear signals of C─C coupling intermediates were observed over pristine W_18_O_49_ (Figure S24, Supporting Information), verifying that atomically‐dispersed Cu(I) sites play a critical role in the C─C coupling process. Based on the in‐situ DRIFTS detection of *CO and *CHO─CO intermediates over 3.6‐Cu_1_/W_18_O_49_ photocatalyst, we propose a possible pathway for the reduction of CO_2_ to C_2_H_4_ as follows:

(2) $$*+CO_{2}+e^{-}+H^{+}\to *\mathrm{COOH}$$

(3) $$*\mathrm{COOH}+e^{-}+H^{+}\to *\mathrm{CO}+H_{2}O$$

(4) $$*\mathrm{CO}+e^{-}+H^{+}\to *\mathrm{CHO}$$

(5) $$*\mathrm{CHO}+5e^{-}+5H^{+}\to CH_{4}+H_{2}O$$

(6) ∗CO+∗CO→∗CO−∗CO

(7) $$*\mathrm{CO}-*\mathrm{CO}+e^{-}+H^{+}\to *\mathrm{CHO}-*\mathrm{CO}$$

(8) $$*\mathrm{CHO}-*\mathrm{CO}+7e^{-}+7H^{+}\to C_{2}H_{4}+*+2H_{2}O$$

where * denotes the catalytically active sites To further reveal the underlying mechanism of photocatalytic CO_2_ reduction to C_2_H_4_, DFT calculations were performed. Given the results of XRD, TEM and XAFS measurements, four oxygen‐coordinated Cu single atoms on the (100) crystal plane of W_18_O_49_ were constructed and refined (Figure S25, Supporting Information). As a prerequisite for *CO intermediates, the formation barrier of *COOH was first calculated (Figure S26, Supporting Information). Compared to pristine W_18_O_49_, it can be observed that the introduction of Cu single atoms significantly reduces the formation barrier of *COOH intermediate. As demonstrated in in‐situ DRIFTS, the coupling of two *CO intermediates is the key step of the reaction toward C_2_H_4_ formation. To investigate this process, we studied the adsorption capacity of the key *CO intermediates over the 3.6‐Cu_1_/W_18_O_49_ and pristine W_18_O_49_ photocatalysts. First, to determine the possible active sites of the reaction, the Gibbs free energy at different sites of *CO adsorption—a key intermediate of C─C coupling, was investigated (Figure 5c). The adsorption of *CO on Cu sites (Cu─*CO) and W sites (W─*CO) release energy of 1.15 and 0.14 eV, respectively; this indicates more favorable CO adsorption on Cu(I) sites, and is consistent with CO desorption DRIFTS results. Interestingly, W_18_O_49_ and Cu_1_/W_18_O_49_ exhibit completely different thermodynamic processes when a second CO is adsorbed on the neighboring sites. The adsorption of another *CO on the adjacent W atom in pristine W_18_O_49_ (W─*CO, W─*CO) requires overcoming a high energy barrier (0.75 eV). In contrast, the energy barrier for the adsorption of another *CO on the adjacent W atom with Cu atom (Cu─*CO, W─*CO) is only −0.02 eV, which is nearly a spontaneous process. Consequently, it is thermodynamically unfavorable to produce two adjacent *CO intermediates at W sites in pristine W_18_O_49_, however, the introduction of Cu(I) sites modulate the adsorption of *CO intermediates on W sites and favor the generation of two adjacent *CO on the asymmetric Cu─W dual sites. This result also indicates that the W sites in the Cu─W dual sites can also have a stronger adsorption of *CO. The formation of double *CO adsorption on Cu_1_/W_18_O_49_ surface can increase the probability of C─C coupling and improve the corresponding reaction kinetically. The generation of *CO─*CO from CO* (2*CO→*CO─CO) is usually considered a crucial step in the formation of C_2+_ compounds.^[^ ^59^, ^60^ ^]^ Notably, the formation energy of *CO─*CO on Cu_1_/W_18_O_49_ (0.50 eV) is lower compared to the pristine W_18_O_49_ (0.57 eV), indicating that the asymmetric Cu─W dual sites lower the thermodynamic energy barrier of *CO coupling and promote the formation of C─C bonds.

The full‐path Gibbs free energy changes (Figure 5d) were then calculated for the generation of C_2_H_4_ on W_18_O_49_ and Cu_1_/W_18_O_49_, respectively. With corresponding structural models (Figures S27 and S28, Supporting Information), it is clear that *CO intermediate on W_18_O_49_ prefers to desorb as CO product (0.34 eV), rather than combine together to form *COCO (1.32 eV). By contrast, the overall free energy of C_2_H_4_ generation process at the asymmetric Cu─W dual sites is a downhill path, demonstrating the thermodynamic advantage of the Cu─W dual sites toward C_2_H_4_ formation. To further elucidate the role of Cu─W dual sites in the C─C coupling process, DFT calculations on transition state barriers were carried out. As shown in Figure 5e, Cu_1_/W_18_O_49_ exhibits a lower transition state barrier (0.66 eV) for C─C coupling process compared to pristine W_18_O_49_ (0.83 eV), indicating the facilitating role of the Cu─W asymmetric dual sites in this crucial rate‐determining step. In brief, DFT calculations unravel that CO adsorption is more favorable at the asymmetric Cu─W dual sites, which lower the energy barrier of C─C coupling and promote the photoreduction of CO_2_ to C_2_H_4_.

## 3 Conclusion

We have successfully designed and prepared stabilized Cu(I)‐containing asymmetric Cu─W dual sites, that is, Cu(I) single atoms modified W_18_O_49_ nanowires photocatalyst, for selective photocatalytic CO_2_ reduction to C_2_H_4_. Impressively, under light irradiation, the optimal Cu_1_/W_18_O_49_ photocatalyst exhibits C_2_H_4_ production with concurrent high activity and selectivity. Through interconversion of W(V) and W(VI) in W_18_O_49_, Cu(I) single atoms maintain their stability during photocatalytic process. Compared to pristine W_18_O_49_, the introduction of Cu(I) single atoms stabilize *CO intermediates and improve the adsorption of *CO on adjacent W atoms in Cu_1_/W_18_O_49_. As a consequence, the as‐formed asymmetric Cu─W dual sites significantly reduce the energy barrier of C─C coupling, thus leading to the high selectivity of C_2_H_4_ product from photocatalytic CO_2_ reduction over Cu_1_/W_18_O_49_. This work provides new insight into design of rational active sites at the atomic level for CO_2_ photoreduction to C_2+_ products, and may shed some light on future selective artificial photosynthesis.

## Conflict of Interest

The authors declare no conflict of interest.

## Supporting information

Supporting Information

## Data Availability

The data that support the findings of this study are available in the supplementary material of this article.
