# Supplementary material for: Asymmetric Cu(I)─W Dual‐Atomic Sites Enable C─C Coupling for Selective Photocatalytic CO2 Reduction to C2H4
Source: Adv Sci (Weinh). 2024 Apr 26;11(28):2401933. doi: 10.1002/advs.202401933 (PMC11267401; doi:10.1002/advs.202401933)
Supplement: Supplementary file 1 — Supporting Information [file ADVS-11-2401933-s001.pdf]

ADVANCED SCIENCE
Open Access

Supporting Information

for *Adv. Sci.*, DOI 10.1002/advs.202401933

Asymmetric Cu(I)—W Dual-Atomic Sites Enable C—C Coupling for Selective Photocatalytic CO_2 Reduction to C_2H_4

Yuyin Mao, Minghui Zhang, Guangyao Zhai, Shenghe Si, Dong Liu, Kepeng Song, Yuanyuan Liu, Zeyan Wang, Zhaoke Zheng, Peng Wang, Ying Dai, Hefeng Cheng and Baibiao Huang**

Supporting Information

Asymmetric Cu(I)–W Dual-Atomic Sites Enable C–C Coupling for Selective Photocatalytic CO_2 Reduction to C_2H_4

Yuyin Mao, Minghui Zhang, Guangyao Zhai, Shenghe Si, Dong Liu, Kepeng Song, Yuanyuan Liu, Zeyan Wang, Zhaoke Zheng, Peng Wang, Ying Dai, Hefeng Cheng, and Baibiao Huang**

Experimental Section

Chemicals and Materials

Anhydrous ethanol (C_2H_5OH) was obtained from Beijing Chemical Corporation. Tungsten hexachloride (WCl_6) and copper chloride ($CuCl_2·2H_2O$) were purchased from Macklin Co. All chemical reagents were used as received without further purifications. Deionized water was made by the Millipore system and used in all experiments.

Synthesis of $W_{18}O_{49}$ Nanowires

In a typical synthetic procedure, 50 mg of WCl_6 was dissolved in 30 mL ethanol to obtain a bright yellow solution. It was then transferred to a 50 mL Teflon-lined stainless steel autoclave and heated at 200 °C for 12 h. Finally, the product was centrifuged and washed with deionized water for several times, and dried under vacuum at 60 °C overnight.

Synthesis of *x*-$Cu_1/W_{18}O_{49}$ Nanowires

In a typical synthetic procedure, 50 mg of WCl_6 was dissolved in 30 mL ethanol to obtain a bright yellow solution. To the bright yellow solution, a certain amount (0.1 mL, 0.2 mL, 0.25 mL, 0.4 mL, 0.5 mL) of $CuCl_2·2H_2O$ solution (3 mg mL^{-1}) was added, which was further stirred for 10 min. It was then transferred to a 50 mL Teflon-lined stainless steel autoclave and heated at 200 °C for 12 h. Finally, the product was centrifuged and washed with deionized water for several times, and dried under vacuum at 60 °C overnight.

Synthesis of Cu(II)/WO_3

To prepare Cu(II)/WO_3, 3.6-$Cu_1/W_{18}O_{49}$ samples was calcined in air at 450 °C for 4 h.

Characterizations

The morphology and microstructure of the samples were characterized by SEM (Hitachi S-4800) and HRTEM (JEOL JEM-2100 F). HAADF-STEM images and elemental mapping were collected at 300 kV on a ThermoFisher Scientific Spectra 300 scanning transmission electron microscope equipped with a Super-X EDS detector system. The phase and structural information of the samples were detected by XRD (Bruker AXS D8 diffractometer). To ascertain the chemical composition and valence state, the samples were studied by XPS (Thermo Fisher Scientific Escalab 250 spectrometer). UV-vis absorption spectra were recorded by a Shimadzu UV 2550 spectrophotometer. The content of Cu in the x-$Cu_1/W_{18}O_{49}$ samples was measured by ICP-MS on a PerkinElmer NexION 1000G instrument. The oxygen vacancies were recorded by EPR (Bruker EMXplus). CO temperature-programmed desorption (CO-TPD) is tested on AutoChem1 II 2920.

XAFS Measurements and Analysis

XAFS analyses were performed with Si (111) crystal monochromators at the BL14W beamline at the Beijing Synchrotron Radiation Facility. Before the analysis at the beamline, samples were placed into aluminum sample holders and sealed using Kapton tape film. Particular care was taken to minimize the beam-induced oxidation of the samples by placing the sample stands filled with pristine and reacted samples in a nitrogen-filled glove box for 6 hours before transferring them to zippered bags in this glove box. The XAFS spectra were recorded at room temperature using a 4-channel Silicon Drift Detector (SDD) Bruker 5040. The EXAFS spectra were recorded in fluorescence mode. The acquired EXAFS data were processed according to the standard procedures using the Athena program implemented in the IFEFFIT software packages. The normalized EXAFS spectra were obtained by setting the pre-edge and post-edge to 0 and 1, respectively. Then, the χ (k) data were Fourier transformed into real (R) space by a Hanning window with sill size of 1.0 $Å^{-1}$ to separate the EXAFS contributions from the distinct coordination shells. To obtain quantitative structural

parameters around Cu atoms, least-squares curve parameter fitting was performed using the ARTEMIS module of the IFEFFIT software packages.

Photocatalytic CO_2 Reduction

For the photocatalytic CO_2 reduction reaction, 5 mg of catalyst was dispersed in 500 μL of aqueous solution and then dropped onto quartz fiber paper (diameter 25 mm). Then it was dried in a vacuum oven at 60°C. The quartz fiber paper containing the catalyst was placed at the bottom of the reactor and 200 μL of water was added dropwise to the reactor. This was followed by the introduction of CO_2 feeding gas for 30 min to expel the air. A 300W Xe lamp (Beijing China Education Au-light Co.) with a light intensity of 453 mW cm^{-2} was used as the light source. After the reaction, the generated products were quantified by a FuLi GC9790 gas chromatography (GC).

The selectivity of C_2H_4 is calculated as follows: Selectivity of

$$Selectivity\ of\ C_2H_4 = \frac{n_{C_2H_4} * 12}{n_{C_2H_4} * 12 + n_{CH_4} * 8 + n_{CO} * 2} \times 100\% \cdots\cdots\cdots\cdots\cdots (1)$$

where $n_{C_2H_4}$, n_{CH_4} and n_{CO} represent the yield of C_2H_4, CH_4 and CO during the photocatalytic reactions, respectively.

^{13}C Isotope Labeling Experiments

In the ^{13}C isotope labeling experiment, 5 mg of catalyst was dispersed in 500 μL of aqueous solution and then dropped onto quartz fiber paper (diameter 25 mm). Then it was dried in a vacuum oven at 60 °C. The quartz fiber paper containing the catalyst was placed at the bottom of the reactor and 200 μL of water was added dropwise to the reactor. This was followed by the introduction of $^{13}CO_2$ feeding gas into a quartz reactor (50 mL) previously vented by argon. A 300W Xe lamp was used as the light source, and the final products were detected by Shimazu GCMS-QP2020.

In-situ NAP-XPS Measurements

In-situ NAP-XPS measurements were performed at the photoemission end-station at beamline BL11U in the National Synchrotron Radiation Laboratory (NSRL) in Hefei, China. The end station is composed of three chambers—an analysis chamber, a preparation chamber, a load-lock chamber. The catalyst was dropcast onto a clear

silicon wafer, and dried at room temperature. High-purity CO_2 (99.9999%) was introduced into the analysis chamber with the partial pressure maintained at 0.25 mbar. Then, the sample was transferred to the analysis chamber for XPS measurement without exposing it to air. A Xe lamp source (PLS-SXE300D, Beijing Perfectlight) was used as a light source.

In-situ DRIFTS Measurements

In-situ DRIFTS measurements were performed using a Thermo Scientific Nicolet iS50 FT-IR with a liquid-nitrogen-cooled HgCdTe detector. Each spectrum was recorded by averaging 64 scans at a resolution of 4 cm^{-1}. The sample was held in a custom-fabricated IR reaction chamber which was specifically designed to examine highly scattered powder samples in diffuse reflection mode. The chamber was sealed with two ZnSe windows. The spectra were collected under dark conditions or after a certain irradiation time using a 300 W Xe lamp (PerfectLight, China). The spectra were obtained by subtracting the background from the spectra of samples.

DFT Calculations

DFT calculations were performed using the quantum espresso (QE) based on the pseudopotential plane wave (PPW) method. The Perdew-Bueke-Ernzerhof (PBE) functional was used to describe exchange-correlation effects of electrons. We have chosen the projected augmented wave (PAW) potentials to describe the ionic cores and take valence electrons into account using a plane wave basis set with a energy cutoff of 400 eV in all relaxation processes. The convergence criteria were set to 10^{-5} eV for the energy and −0.001 eV/Å for the force. The k-point meshes were set of 2 × 3 × 1, 3 × 3 × 1, 7 × 7 × 1 for geometry optimization, electronic self-consistent, and the electronic structure calculation, respectively. All constructions possess larger than 15 Å vacuum region to minimize the interactions between adjacent image cells.

According to the computational hydrogen electrode (CHE) model proposed by Nørskov. Herein, the free energy ($H^+ + e^-$) of the electron-proton pair can be regarded as the chemical potential of 1/2 gaseous H_2 in equilibrium (0 V for standard hydrogen electrode). The Gibbs free energy between each electrocatalytic reaction step was

obtained by the following equation:

$$\Delta G = \Delta E + \Delta E_{\mathrm{ZPE}} - T\Delta S \quad (2)$$

Where ΔE, ΔE_{ZPE} and ΔS represent the difference of adsorption energy, zero-point energy, and entropy at 298.15 K of the reaction intermediate on the substrate, respectively. The zero-point energies and entropies of the reaction intermediate were calculated through vibration frequencies, in which we fix the substrate and allow the absorb rate vibrational modes to be computed.

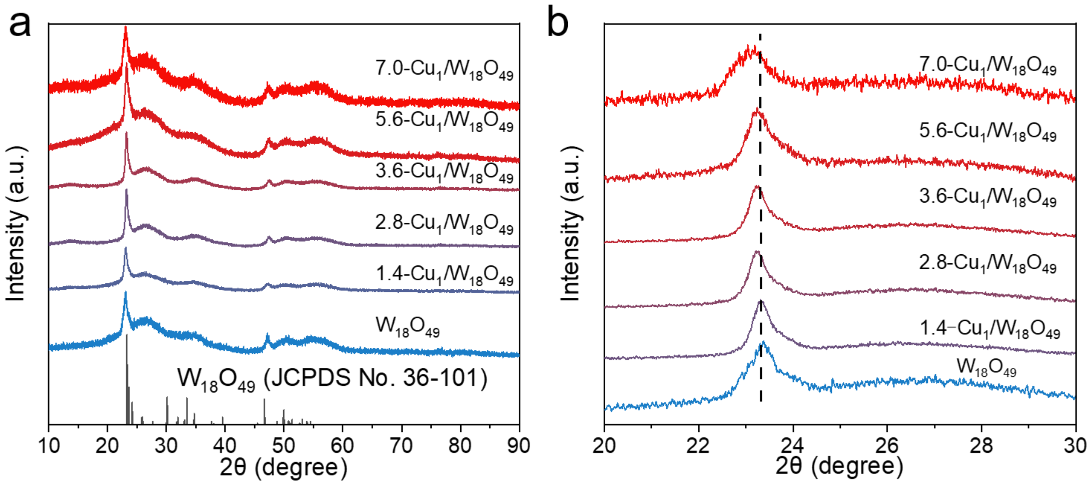

Figure S1. XRD patterns of pristine $W_{18}O_{49}$ and x-$Cu_1/W_{18}O_{49}$ (x = 1.4, 2.8, 3.6, 5.6, 7.0).

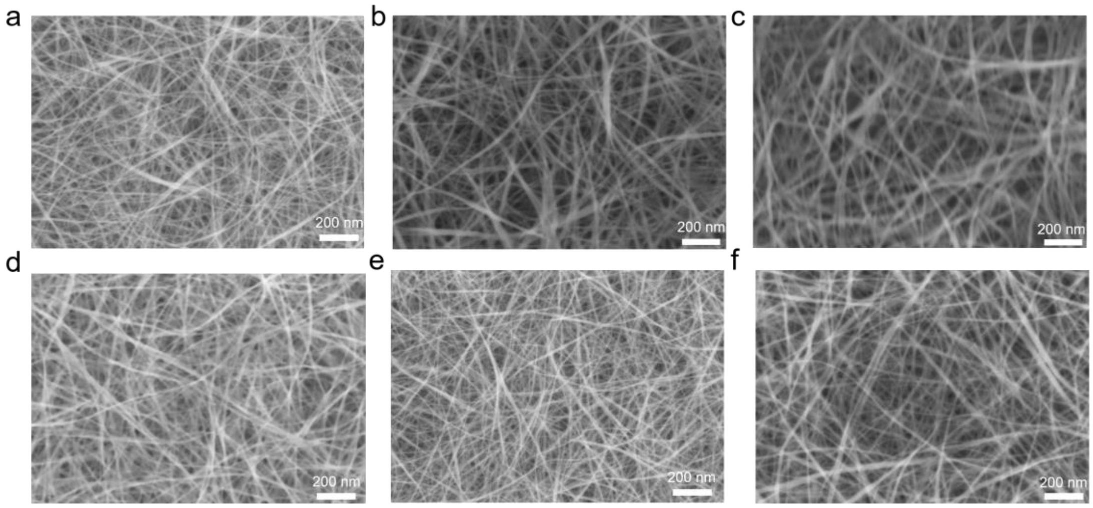

Figure S2. Morphological characterizations of $W_{18}O_{49}$ and x-$Cu_1/W_{18}O_{49}$. SEM images of (a) $W_{18}O_{49}$, (b) 1.4-$Cu_1/W_{18}O_{49}$, (c) 2.8-$Cu_1/W_{18}O_{49}$, (d) 3.6-$Cu_1/W_{18}O_{49}$, (e) 5.6-

$Cu_1/W_{18}O_{49}$, and (f) 7.0-$Cu_1/W_{18}O_{49}$.

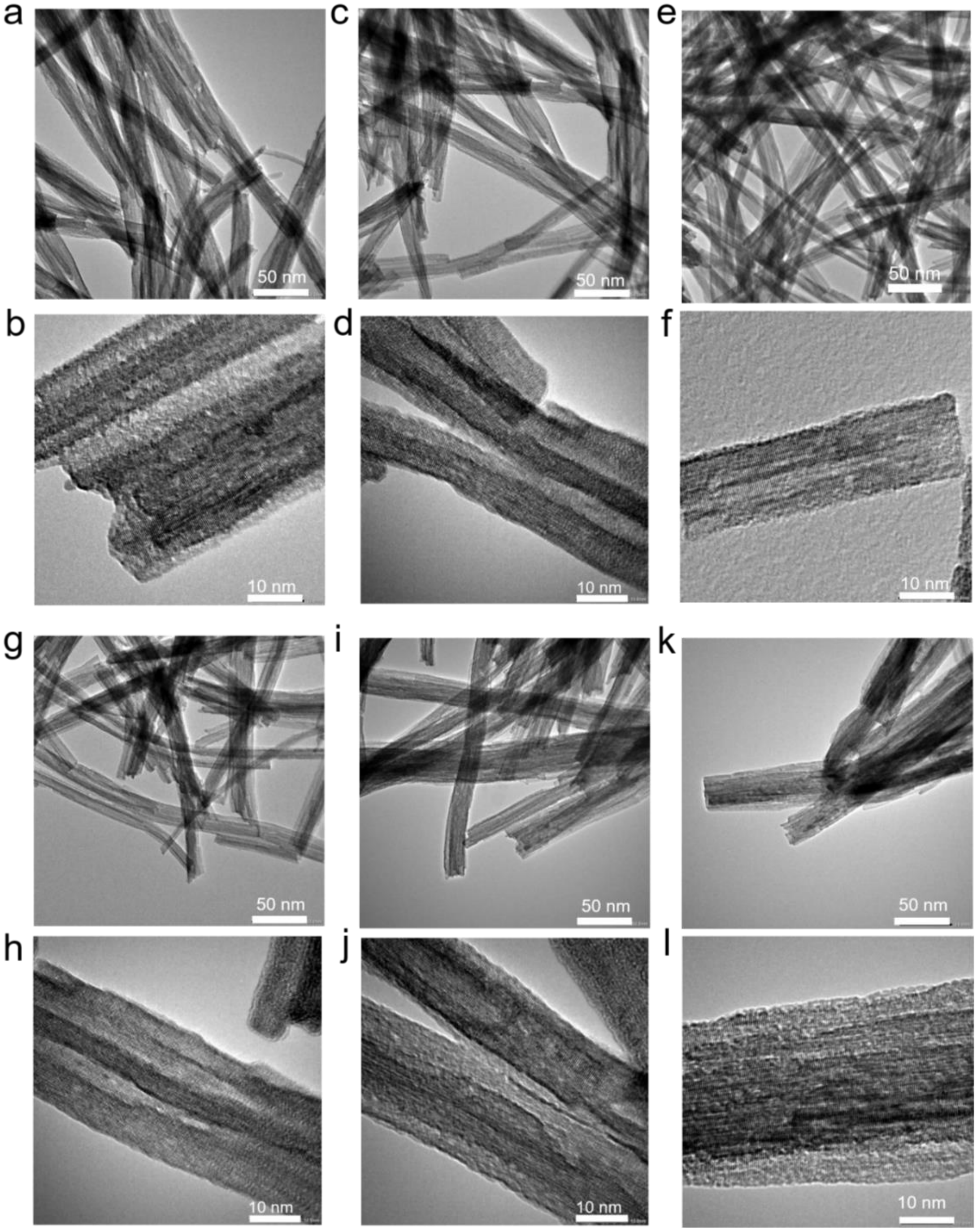

Figure S3. Morphological characterizations of $W_{18}O_{49}$ and x-$Cu_1/W_{18}O_{49}$. TEM and HRTEM images of (a, b) $W_{18}O_{49}$, (c, b) 1.4-$Cu_1/W_{18}O_{49}$, (e, f) 2.8-$Cu_1/W_{18}O_{49}$, (g, h) -3.6-$Cu_1/W_{18}O_{49}$, (i, j) 5.6-$Cu_1/W_{18}O_{49}$, and (k, l) 7.0-$Cu_1/W_{18}O_{49}$.

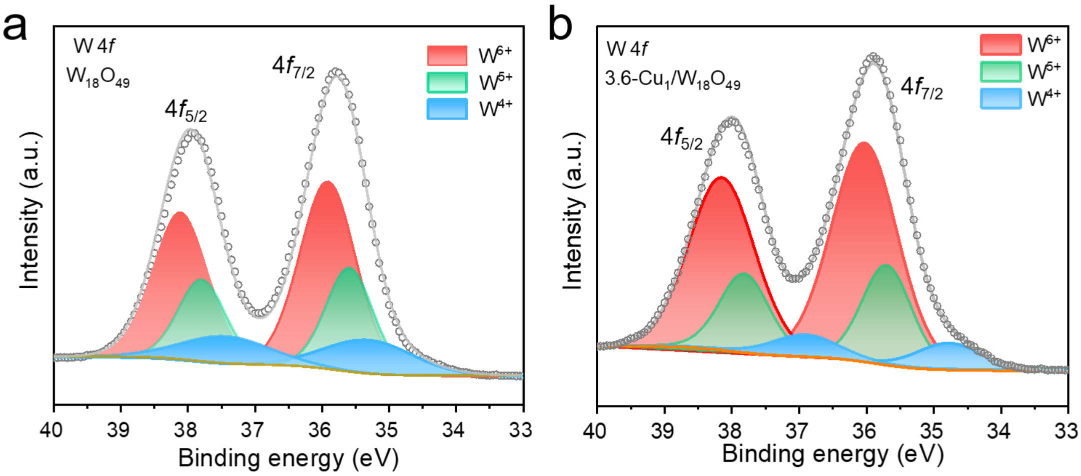

Figure S4. W 4*f* XPS spectra of (a) $W_{18}O_{49}$ and (b) 3.6-$Cu_1/W_{18}O_{49}$.

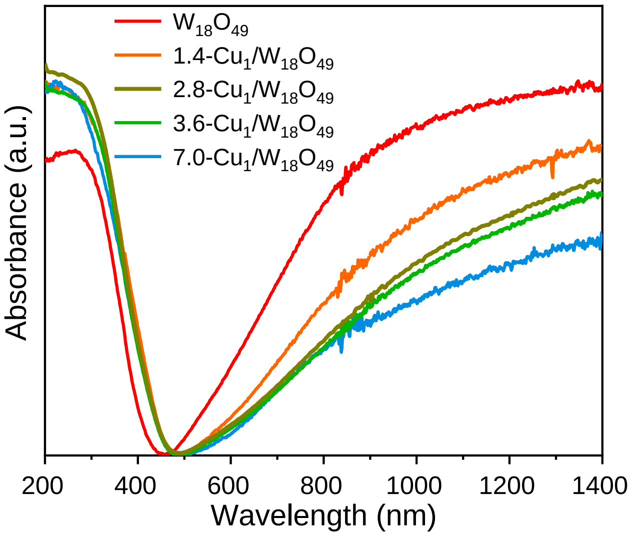

Figure S5. UV-vis diffuse reflectance spectra of $W_{18}O_{49}$ and *x*-$Cu_1/W_{18}O_{49}$ samples.

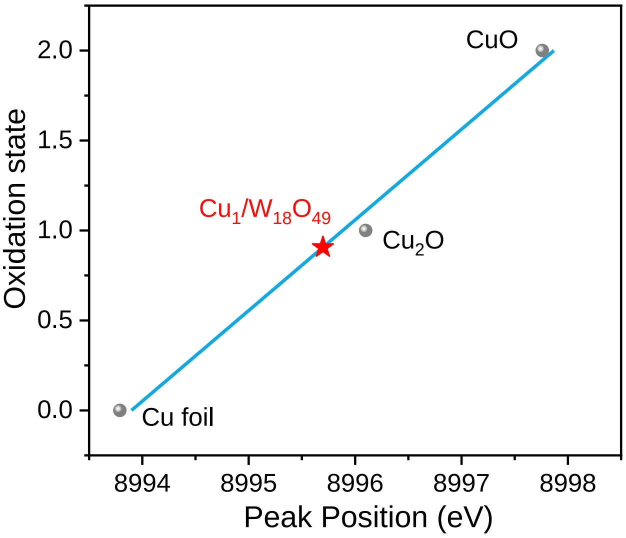

Figure S6. The linear combination fitting for the position of peak in XANES curves.

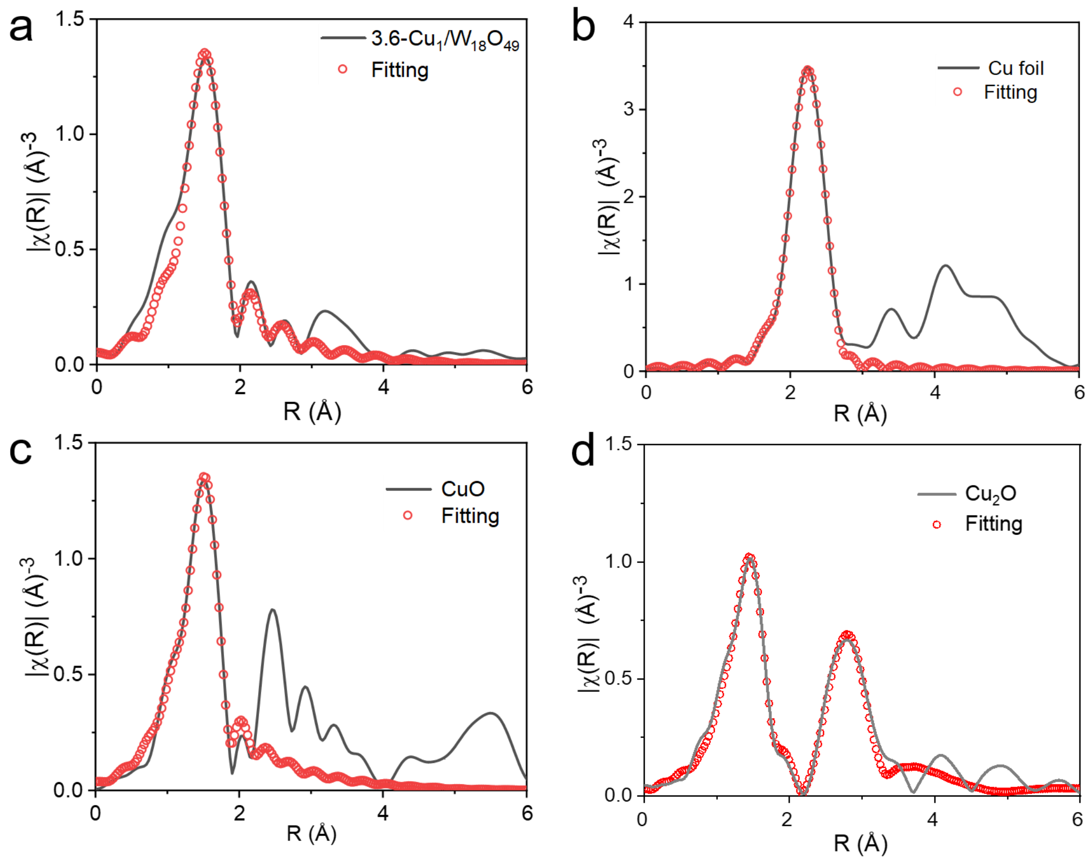

Figure S7. EXAFS data and the fitting plots for (a) 3.6-$Cu_1/W_{18}O_{49}$, (b) Cu foil, (c) CuO and (d) Cu_2O in R space.

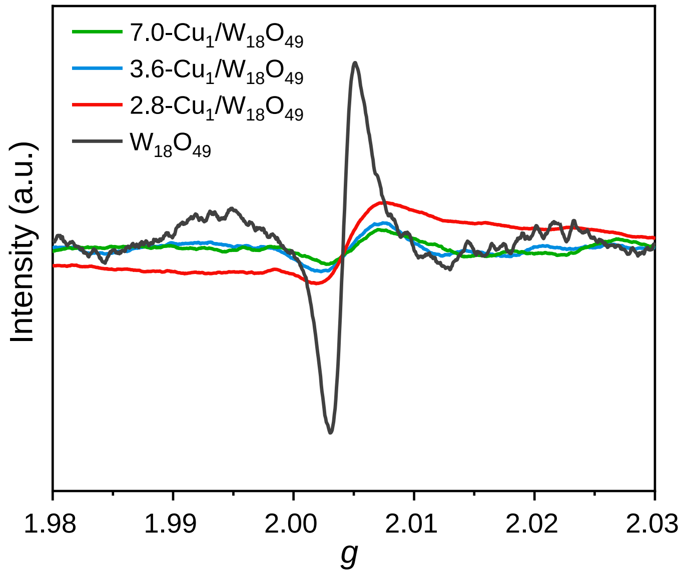

Figure S8. ESR spectra of $W_{18}O_{49}$, 2.8-$Cu_1/W_{18}O_{49}$, 3.6-$Cu_1/W_{18}O_{49}$, and 7.0-$Cu_1/W_{18}O_{49}$.

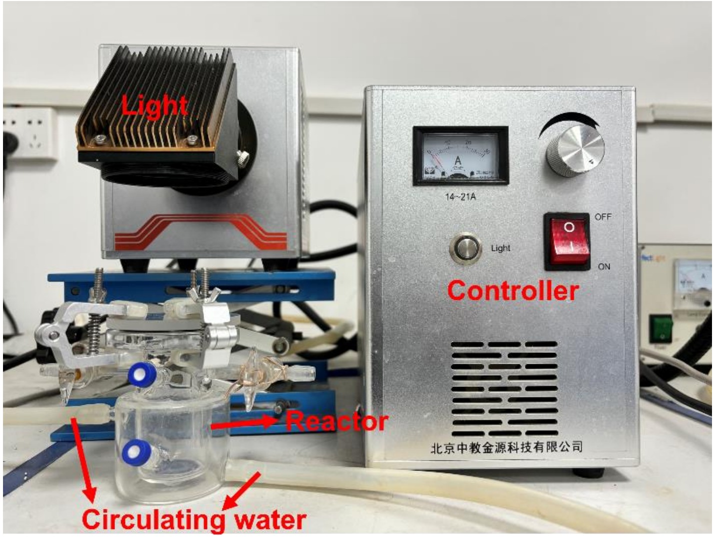

Figure S9. Image of the reaction device for photocatalytic CO_2 reduction.

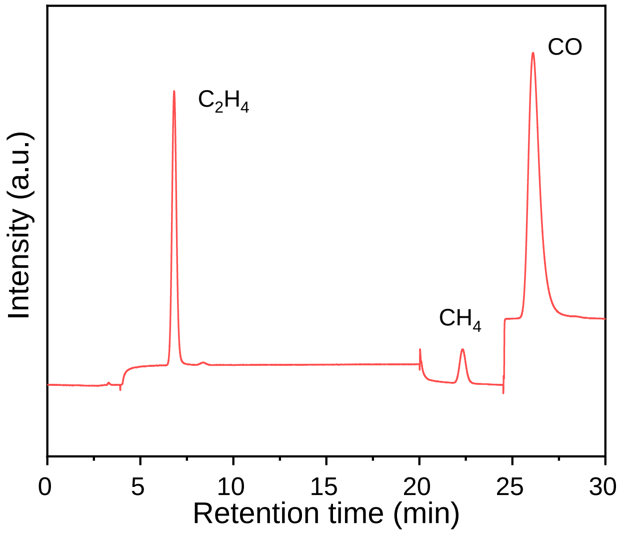

Figure S10. GC spectrum of gas products over 3.6-$Cu_1/W_{18}O_{49}$.

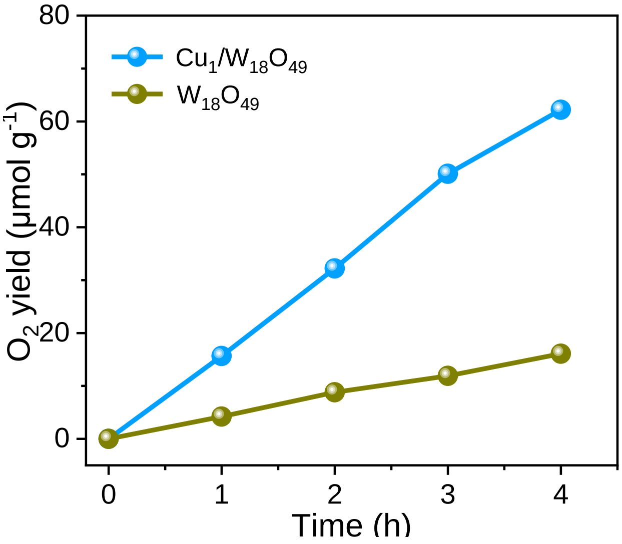

Figure S11. The yield rate of O_2 as a function of reaction time over $W_{18}O_{49}$ and 3.6-$Cu_1/W_{18}O_{49}$.

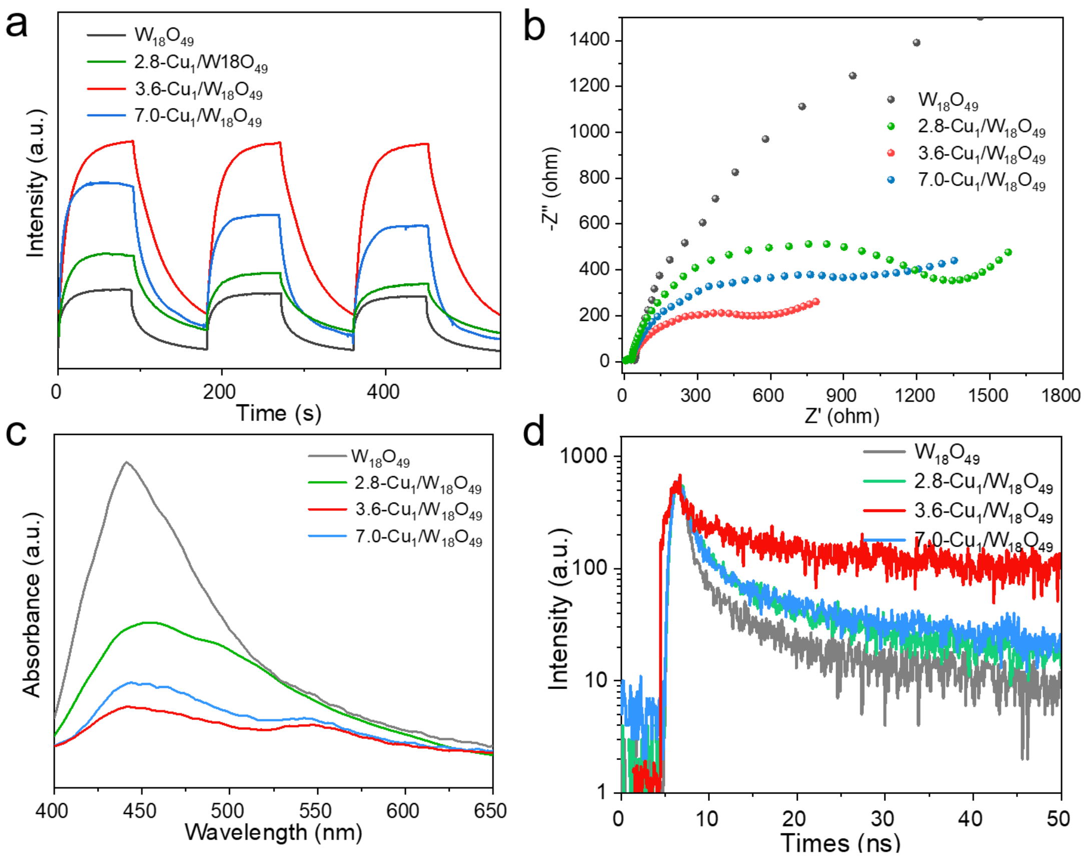

Figure S12. Charge carriers transfer kinetics determination. (a) Transient photocurrent responses of $W_{18}O_{49}$, 2.8-$Cu_1/W_{18}O_{49}$, 3.6-$Cu_1/W_{18}O_{49}$ and 7.0-$Cu_1/W_{18}O_{49}$. (b) EIS Nyquist plots. (c) Steady-state photoluminescence spectroscopy. (d) TRPL

spectroscopy. Among the four samples, 3.6-$Cu_1/W_{18}O_{49}$ exhibits the highest photocurrent intensity, the smallest charge transfer resistance in electrochemical impedance spectrum (EIS) of Nyquist plot, the lowest PL intensity, and the longest average lifetime of charge carriers, verifying its superior separation capacity of photogenerated carriers to other samples.

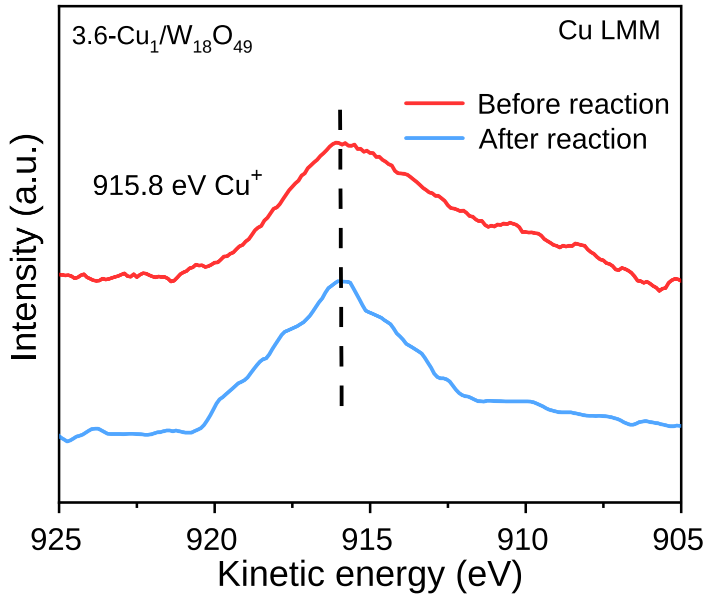

Figure S13. Cu LMM AES of 3.6-$Cu_1/W_{18}O_{49}$ before and after reaction.

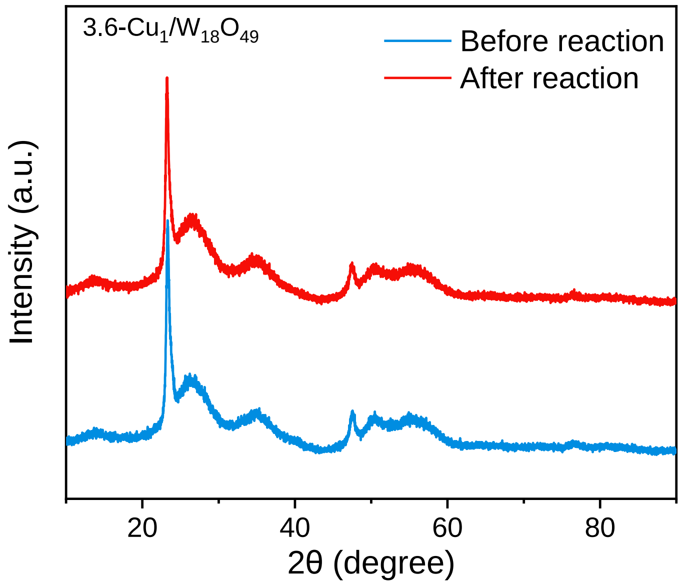

Figure S14. XRD patterns of 3.6-$Cu_1/W_{18}O_{49}$ before and after reaction.

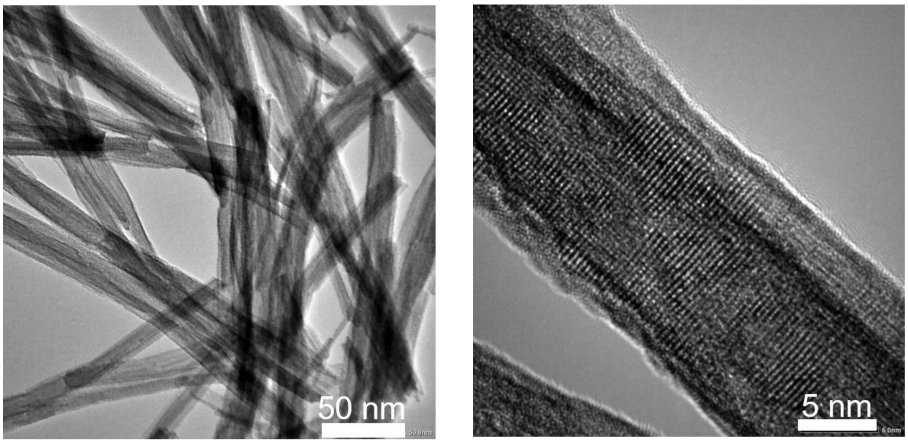

Figure S15. TEM and HRTEM images of 3.6-$Cu_1/W_{18}O_{49}$ after recycling test.

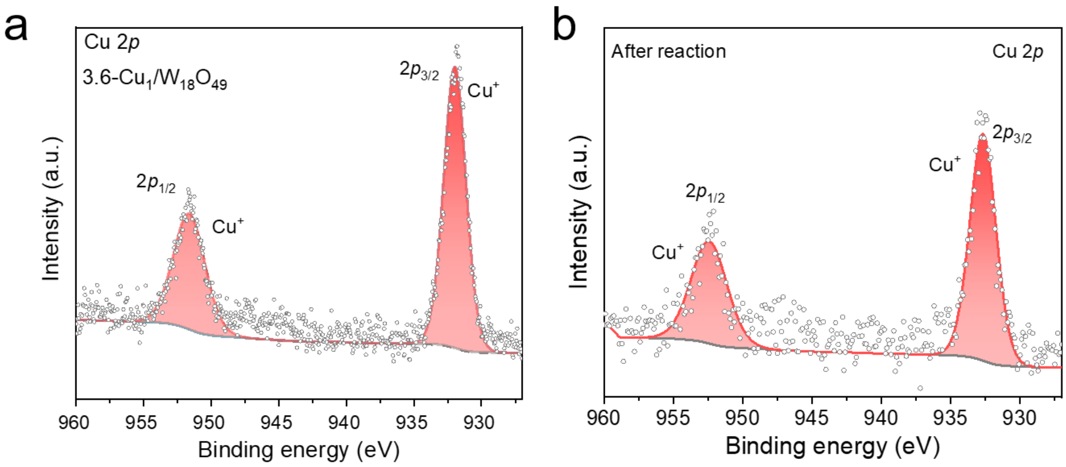

Figure S16. Cu 2*p* XPS spectra of (a) 3.6-$Cu_1/W_{18}O_{49}$ and (b) after reaction.

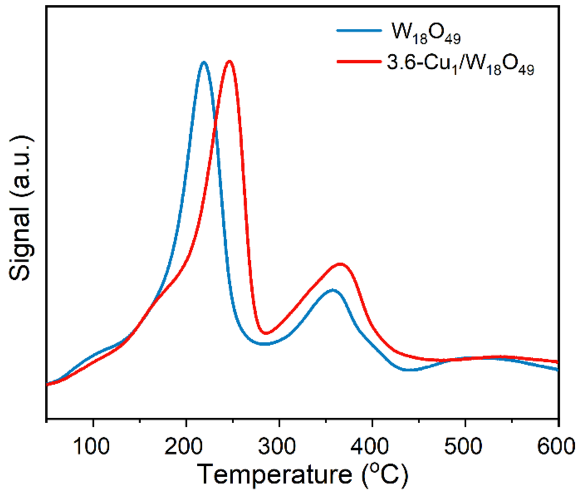

Figure S17. CO-TPD profiles of $W_{18}O_{49}$ and 3.6-$Cu_1/W_{18}O_{49}$ samples.

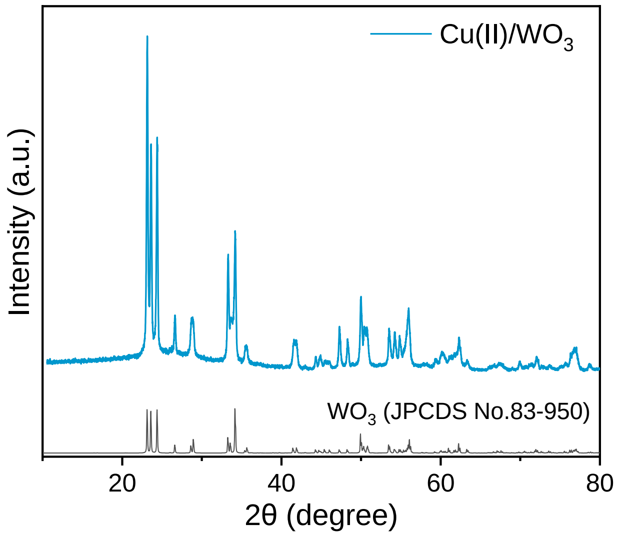

Figure S18. XRD pattern of the as-prepared Cu(II)/WO_3.

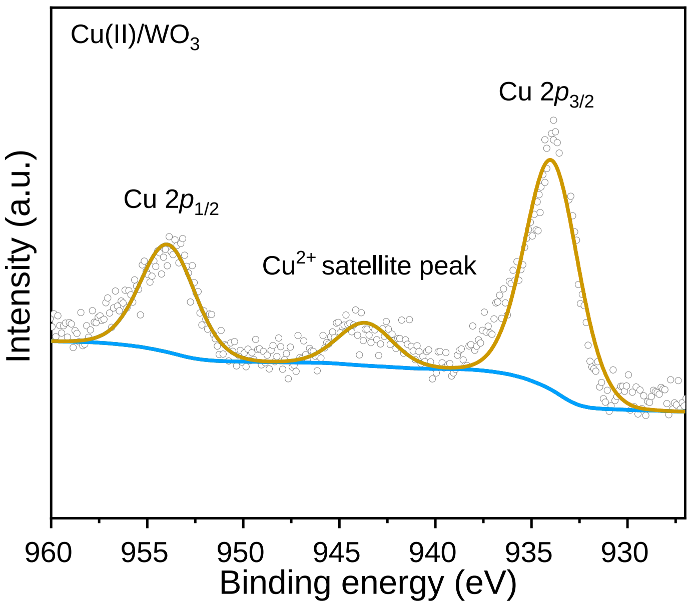

Figure S19. Cu 2*p* XPS spectra of the as-prepared Cu(II)/WO_3.

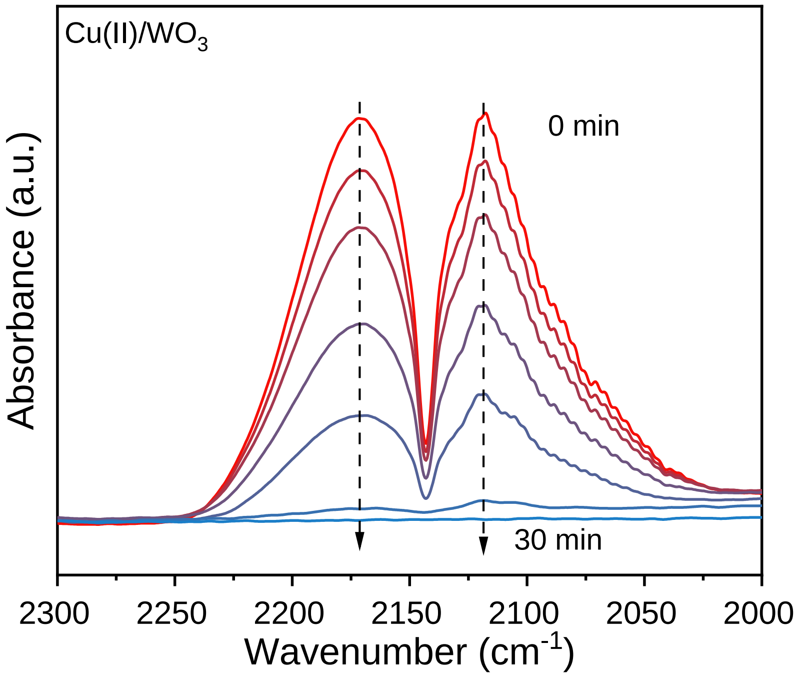

Figure S20. In-situ DRIFT spectra obtained during CO desorption on Cu(II)/WO_3.

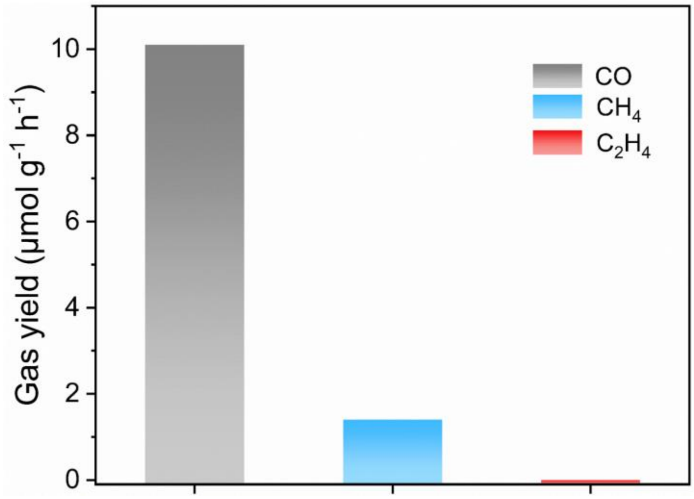

Figure S21. The products yield rate over Cu(II)/WO_3 in photocatalytic CO_2 reduction.

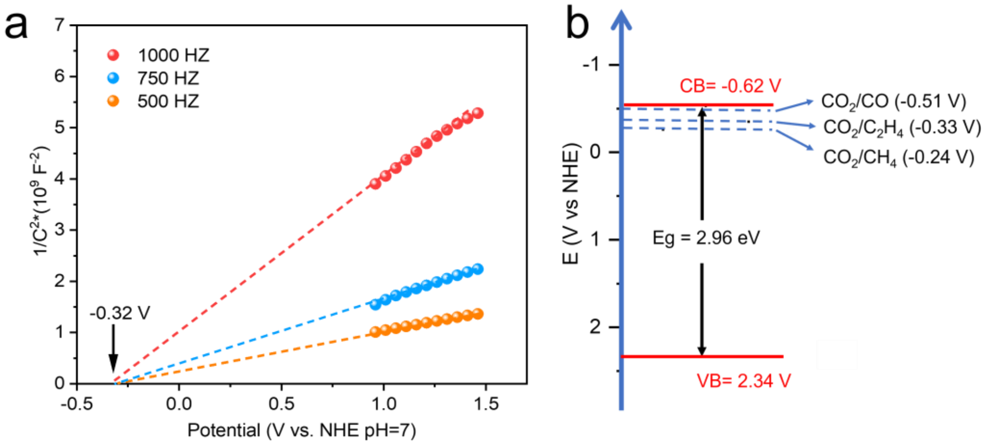

Figure S22. (a) Mott–Schottky plots of 3.6-Cu_1/$W_{18}O_{49}$. (b) Schematic illustration for band structures of 3.6-Cu_1/$W_{18}O_{49}$.

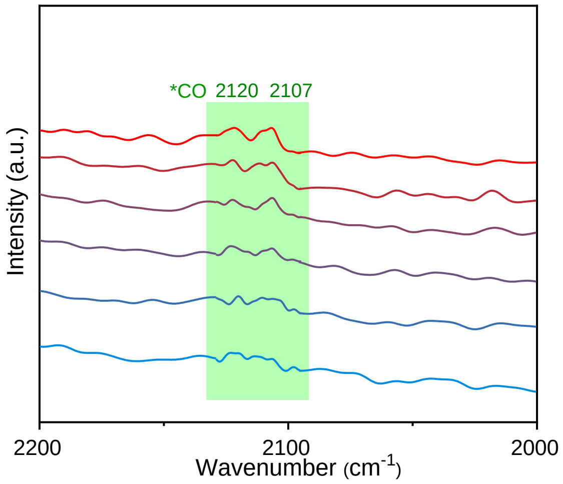

Figure S23. Magnified region of *CO intermediates in in-situ DRIFTS of 3.6-$Cu_1/W_{18}O_{49}$.

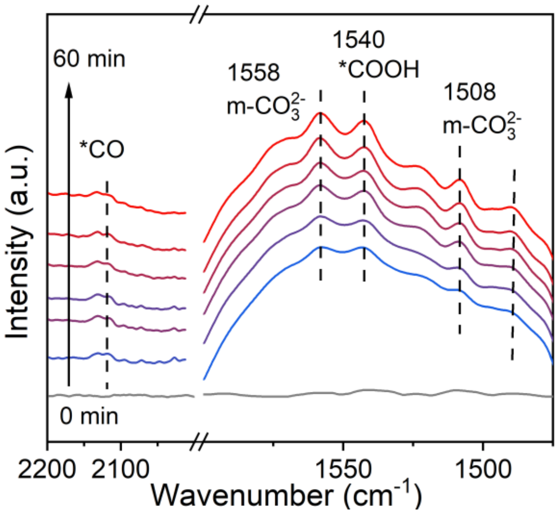

Figure S24. In-situ DRIFT spectra over pristine $W_{18}O_{49}$ under CO_2 feeding gas condition.

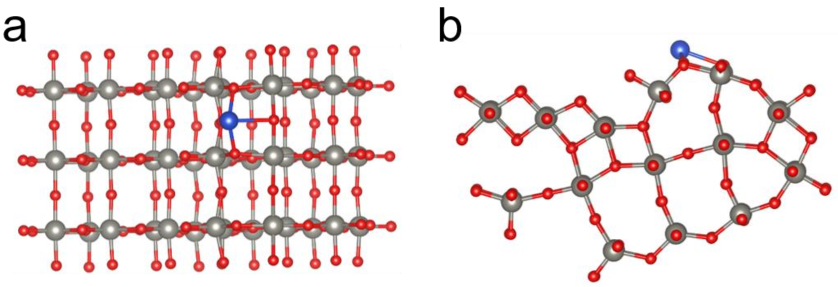

Figure S25. The optimized configurations of $Cu_1/W_{18}O_{49}$. (a) Top view of $Cu_1/W_{18}O_{49}$. (b) Side view of $Cu_1/W_{18}O_{49}$. The blue, grey, and red spheres represent Cu, W, and O atoms, respectively.

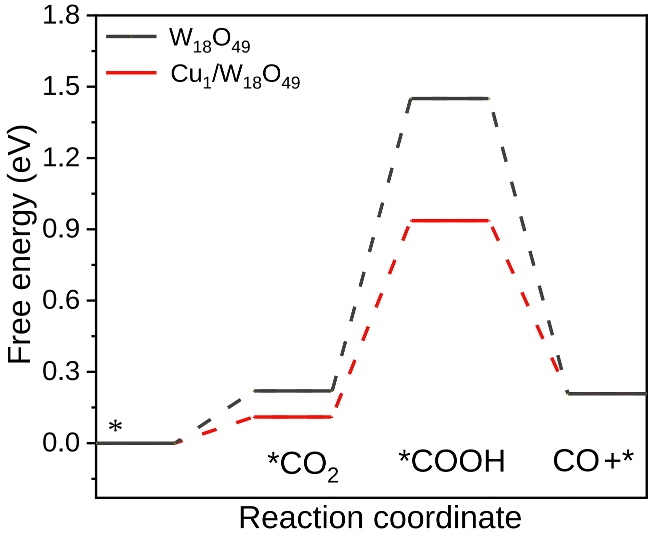

Figure S26. The energy barriers for *COOH formation on $Cu_1/W_{18}O_{49}$ and $W_{18}O_{49}$.

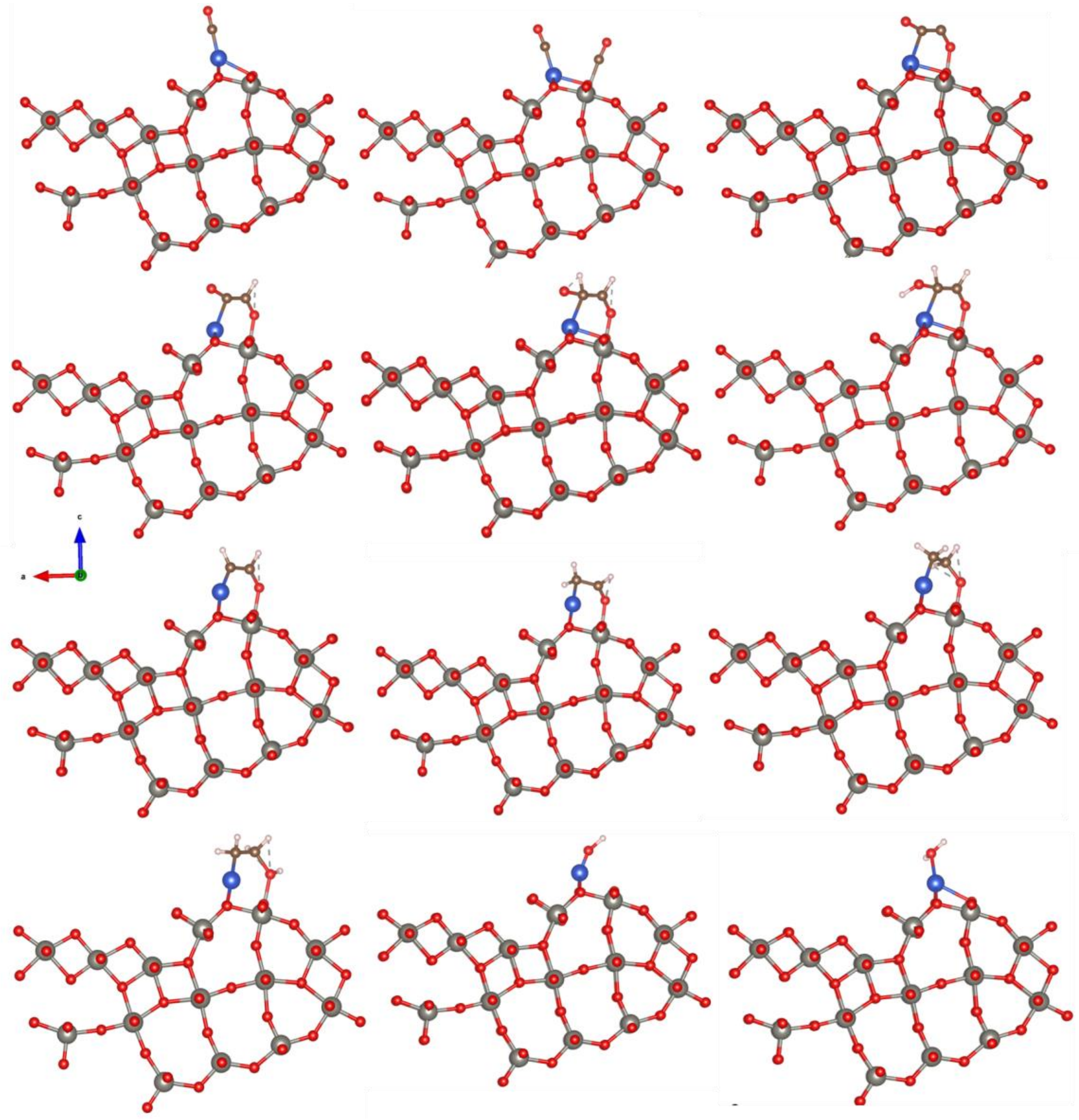

Figure S27. The side views of atomic structures of reaction intermediates on $Cu_1/W_{18}O_{49}$ for C_2H_4 formation reaction path. Blue ball: Cu, gray ball: W, red ball: O, brown ball: C and cyan ball: H.

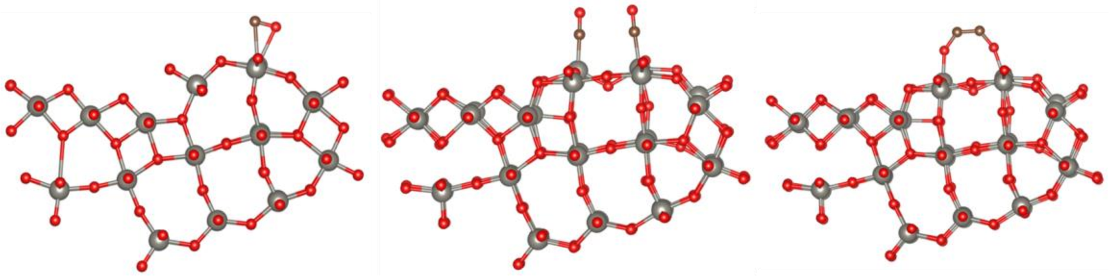

Figure S28. The side views of atomic structures of reaction intermediates on $W_{18}O_{49}$ for *CO coupling. Gray ball: W, red ball: O, brown ball: C and cyan ball: H.

Table S1. The actual content of Cu measured by ICP-MS.

Samples	Theoretical content (wt%)	Actual content (wt%)
1.4-$Cu_1/W_{18}O_{49}$	1.4	1.29
2.8-$Cu_1/W_{18}O_{49}$	2.8	2.44
3.6-$Cu_1/W_{18}O_{49}$	3.6	3.12
5.6-$Cu_1/W_{18}O_{49}$	5.6	4.22
7.0-$Cu_1/W_{18}O_{49}$	7.0	5.01

Table S2. The fitted EXAFS data for the samples of 3.6-$Cu_1/W_{18}O_{49}$, Cu foil, Cu_2O and CuO references.

Sample	Path	CN	R(Å)	σ^2(Å^{-2})	R_f
Cu foil	Cu-Cu	12	2.543	0.009	0.4%
CuO	Cu-O	4	1.947	0.004	0.7%
Cu_2O	Cu-O	4	1.927	0.004	0.6%
3.6-$Cu_1/W_{18}O_{49}$	Cu-O<br>Cu-W	3.89(14)<br>0.88(23)	1.922<br>3.272	0.006<br>0.006	1.2%

CN: coordination number; R: interatomic distance; σ^2: Debye-Waller factor.

Table S3. The comparison of C_2H_4 yield rate and selectivity for the 3.6-$Cu_1/W_{18}O_{49}$ photocatalyst with those recently reported Cu(I)-based photocatalysts for CO_2 reduction to C_{2+} products.

Catalyst	Reduction medium	Product yield rate (μmol g^{-1} h^{-1})	Product selectivity	Ref.
**$Cu_1/W_{18}O_{49}$ (I)**	**CO_2+H_2O (g)**	**C_2H_4, 4.9**	**72.8%**	**This work**
CuO_x@p-ZnO (I/II)	CO_2+H_2O (g)	C_2H_4, 2.7	59.8%	1
$Cu_{0.01}$/3DOM-TiO_2 (I)	CO_2+H_2O (L)	C_2H_4, 6.9	58.4%	2
CuACs/PCN (I)	**Ru-bpy** CO_2+TEOA (L)	C_2H_4, 10.1	53.2%	3
PTF-Cu (I)	**Re-bpy** CO_2+TEOA (L)	C_2H_4, 73.2	25%	4

Table S4. The comparison of C_2H_4 yield rate and selectivity for the 3.6-$Cu_1/W_{18}O_{49}$ photocatalyst with those recently reported systems for photocatalytic CO_2 reduction to C_{2+} products.

Catalyst	Reduction medium	C_2 product yield rate (μmol g^{-1} h^{-1})	C_2 product selectivity	Ref.
**$Cu_1/W_{18}O_{49}$**	**CO_2+H_2O (g)**	**C_2H_4, 4.9**	**72.8%**	**This work**
$Cu^{\delta+}$ /CeO_2-TiO_2	CO_2+H_2O (g)	C_2H_4, 4.51	63.9%	5
$CuGaS_2$	CO_2+0.1 M NaOH (L)	C_2H_4, 20.6	75.1%	6
Bi_2S_3@In_2S_3	CO_2+H_2O (g), photo-thermal	C_2H_4, 11.8	90%	7
Zn_2GaO_4	CO_2+H_2O (g), photo-thermal	CH_3COOH, 12.7	66.9%	8
BP/WO_3	CO_2+H_2O (g)	C_2H_4, 11	13.5%	9
Au_1/RP	$KHCO_3$+HCl	C_2H_6, 1.32	96%	10
Cu SAs/UiO-66-NH_2	CO_2+TEOA	C_2H_5OH, 4.22	~	11
$AgInP_2S_6$	CO_2+H_2O (g)	C_2H_4, 26.6	72.9%	12

Table S5. Free energy of CO adsorption at different sites.

Sample	Site	CO adsorption free energy (eV)
$W_{18}O_{49}$	W	−0.14
$Cu_1/W_{18}O_{49}$	W	−0.52
$Cu_1/W_{18}O_{49}$	Cu	−1.15

Supporting References

[1] W. Wang, C. Deng, S. Xie, Y. Li, W. Zhang, H. Sheng, C. Chen, J. Zhao, *J. Am. Chem. Soc.* **2021**, *143*, 2984.

[2] C. Chen, T. Wang, K. Yan, S. Liu, Y. Zhao, B. Li, *Inorg. Chem. Front.* **2022**, *9*, 4753.

[3] W. Xie, K. Li, X.-H. Liu, X. Zhang, H. Huang, *Adv. Mater.* **2023**, *35,* 2208132.

[4] R. Xu, D.-H. Si, S.-S. Zhao, Q.-J. Wu, X.-S. Wang, T.-F. Liu, H. Zhao, R. Cao, Y.-B. Huang, *J. Am. Chem. Soc.* **2023**, *145*, 8261.

[5] T. Wang, L. Chen, C. Chen, M. Huang, Y. Huang, S. Liu, B. Li, *ACS Nano* **2022**, *16*, 2306.

[6] S. Chakraborty, R. Das, M. Riyaz, K. Das, A. K. Singh, D. Bagchi, C. P. P. Vinod, S. C. C. Peter, *Angew. Chem. Int. Ed.* **2023**, *62*, e202216613..

[7] K. Yan, D. Wu, T. Wang, C. Chen, S. Liu, Y. Hu, C. Gao, H. Chen, B. Li, *ACS Catal.* **2023**, *13*, 2302.

[8] J. Zhu, W. Shao, X. Li, X. Jiao, J. Zhu, Y. Sun, Y. Xie, *J. Am. Chem. Soc.* **2021**, *143*, 18233.

[9] W. Gao, X. Bai, Y. Gao, J. Liu, H. He, Y. Yang, Q. Han, X. Wang, X. Wu, J. Wang, F. Fan, Y. Zhou, C. Li, Z. Zou, *Chem. Commun.* **2020**, *56*, 7777.

[10] H. Ou, G. Li, W. Ren, B. Pan, G. Luo, Z. Hu, D. Wang, Y. Li, *J. Am. Chem. Soc.* **2022**, *144*, 22075.

[11] G. Wang, C.-T. He, R. Huang, J. Mao, D. Wang, Y. Li, *J. Am. Chem. Soc.* **2020**, *142*, 19339.

[12] W. Gao, S. Li, H. He, X. Li, Z. Cheng, Y. Yang, J. Wang, Q. Shen, X. Wang, Y. Xiong, Y. Zhou, Z. Zou, *Nat. Commun.* **2021**, *12*, 4747.
